# Interleukin-1 and Interferon-γ Orchestrate β-Glucan-Activated Human Dendritic Cell Programming *via* IκB-ζ Modulation

**DOI:** 10.1371/journal.pone.0114516

**Published:** 2014-12-04

**Authors:** Marco Cardone, Amiran K. Dzutsev, Hongchuan Li, Nicolas Riteau, Franca Gerosa, Kevin Shenderov, Robin Winkler-Pickett, Lisa Provezza, Elena Riboldi, Robert M. Leighty, Selinda J. Orr, Folkert Steinhagen, Mark D. Wewers, Alan Sher, Stephen K. Anderson, Romina Goldszmid, Daniel W. McVicar, Lyudmila Lyakh, Giorgio Trinchieri

**Affiliations:** 1 Cancer and Inflammation Program, Center for Cancer Research, National Cancer Institute, National Institutes of Health, Frederick, Maryland, United States of America; 2 Basic Science Program, Leidos Biomedical Research, Inc., Frederick National Laboratory for Cancer Research, Frederick, Maryland, United States of America; 3 Immunobiology Section, Laboratory of Parasitic Diseases, National Institute for Allergy and Infectious Diseases, National Institutes of Health, Bethesda, Maryland, United States of America; 4 Department of Pathology, University of Verona, Verona, Italy; 5 Data Management Services, Inc., Frederick National Laboratory for Cancer Research, Frederick, Maryland, United States of America; 6 The Ohio State University, Davis Heart and Lung Research Institute, Columbus, Ohio, United States of America; 7 The Trans-NIH Center for Human Immunology, Bethesda, Maryland, United States of America; University of Colorado School of Medicine, United States of America

## Abstract

Recognition of microbial components *via* innate receptors including the C-type lectin receptor Dectin-1, together with the inflammatory environment, programs dendritic cells (DCs) to orchestrate the magnitude and type of adaptive immune responses. The exposure to β-glucan, a known Dectin-1 agonist and component of fungi, yeasts, and certain immune support supplements, activates DCs to induce T helper (Th)17 cells that are essential against fungal pathogens and extracellular bacteria but may trigger inflammatory pathology or autoimmune diseases. However, the exact mechanisms of DC programming by β-glucan have not yet been fully elucidated. Using a gene expression/perturbation approach, we demonstrate that in human DCs β-glucan transcriptionally activates *via* an interleukin (IL)-1- and inflammasome-mediated positive feedback late-induced genes that bridge innate and adaptive immunity. We report that in addition to its known ability to directly prime T cells toward the Th17 lineage, IL-1 by promoting the transcriptional cofactor inhibitor of κB-ζ (IκB-ζ) also programs β-glucan-exposed DCs to express cell adhesion and migration mediators, antimicrobial molecules, and Th17-polarizing factors. Interferon (IFN)-γ interferes with the IL-1/IκB-ζ axis in β-glucan-activated DCs and promotes T cell-mediated immune responses with increased release of IFN-γ and IL-22, and diminished production of IL-17. Thus, our results identify IL-1 and IFN-γ as regulators of DC programming by β-glucan. These molecular networks provide new insights into the regulation of the Th17 response as well as new targets for the modulation of immune responses to β-glucan-containing microorganisms.

## Introduction

Dendritic cells (DCs) are antigen presenting cells (APCs) that sense microorganisms through innate receptors for microbe-associated molecular patterns (MAMPs). Engagement by single or multiple MAMPs of pattern recognition receptors (PRRs), including Toll-like (TLRs), C-type lectin (CLRs), and other receptors programs DCs to initiate an immune response [1]–[3]. Activated DCs link innate to adaptive immunity by secreting immunoregulatory cytokines that polarize CD4^+^ T helper (Th) cell subsets [4], [5]. Adaptive and innate lymphocyte subsets in turn modulate DC differentiation and activation through soluble molecules such as interferons (IFNs) or interleukin (IL)-4 and by direct cellular contact [6]–[11], thus accentuating specific immune responses.

The knowledge of the molecular mechanisms underlying the DC programming upon recognition of MAMPs by innate receptors is important for the understanding of the regulation of immunity. However, despite there being several innate receptor agonists used in immune support supplements or as adjuvant for vaccines, only the mechanisms regulating the DC response upon TLR triggering have been studied in great detail. It is known that TLR signaling induces immediate and early (primary) genes for inflammatory factors such as tumor necrosis factor (TNF) and type I IFN (IFN-I) required for the regulation of late (secondary) genes encoding key immunoregulatory molecules of the response to pathogens and their components [12]–[18]. Conversely, the molecular requirements controlling the DC programming elicited by immunoreceptor tyrosine-based activation motif (ITAM)-signaling CLRs such as Dectin-1 have been poorly investigated.

Dectin-1 is expressed by myeloid cells and is activated by β-glucans *via* the formation of a “phagocytic synapse” [19]. β-glucans are major structural components of the cell wall of fungi and yeasts that occur as (1,3/1,6)-β-linked glucose polymers [20], [21]. Due to their strong immunostimulatory activity, these microbial carbohydrates are now used as immunomodulators in certain immune support supplements. β-glucan stimulation of Dectin-1 enables DCs to induce Th1 and Th17 adaptive immune responses through inflammatory cytokines regulated by NF-κB, which is activated downstream of the spleen tyrosine kinase (Syk)-dependent formation of the CARD9-Bcl10-MALT1 scaffold and Raf-1 [22]–[25]. Mostly, β-glucan programs human monocyte-derived DCs to release high levels of the Th17-polarizing cytokines IL-1β, IL-6, and IL-23 [26], [27], but the exact mechanism has not yet been fully elucidated. IL-1β release is a multistep process requiring transcription of pro-IL-1β and its inflammasome-dependent processing to the mature form [22], [23], [28]. Dectin-1 signaling through Syk activates the NLRP3 inflammasome *via* reactive oxygen species (ROS) and K^+^ efflux, a mechanism required for the defense against fungal infections [29]–[31].

The present study was designed to identify key regulators, and their mechanism of action, of the immunity to β-glucan initiated by human DCs. As reported for TLR ligands, we show that β-glucan also induces early and late immunoregulatory genes. Analysis of the kinetics of gene expression following DC activation by β-glucan predicted the early genes *IL1*, *TNF*, and *IFNB1* to be regulators of the β-glucan-mediated transcriptional response. A perturbation analysis revealed that autocrine/paracrine IL-1 selectively supports the β-glucan-induced programming of human DCs, while TNF and IFN-I modulate the response to both β-glucan and the TLR4-agonist, lipopolysaccharide (LPS), chosen as a comparison for a prototypical TLR stimulation.

TLR-induced activity of NF-κB and other transcription factors (TFs), controlling the expression of key immunomodulatory genes, can be regulated by the Inhibitor of κB-ζ (IκB-ζ) a member of the IκB family induced by MyD88-associated receptors [32]–[36]. IκB-ζ also directly promotes IL-17 production in Th17 cells by cooperating with the TF ROR(γ)T [37].

We now demonstrate that the MyD88-dependent signaling by IL-1 ensures a complete activation of β-glucan-exposed DCs by maintaining high levels of nuclear IκB-ζ that are required for the optimal expression of late genes encoding cell adhesion and migration mediators, antimicrobial molecules, and Th17-polarizing factors. Conversely, IFN-γ reprograms IL-17-inducing DCs activated by β-glucan into IFN-γ/IL-22-inducing APCs by affecting their ability to induce IL-1/IκB-ζ and downstream immunoregulators including Th17-promoting factors.

## Results

### β-glucan regulates gene expression in human DCs

**Figure 1:**
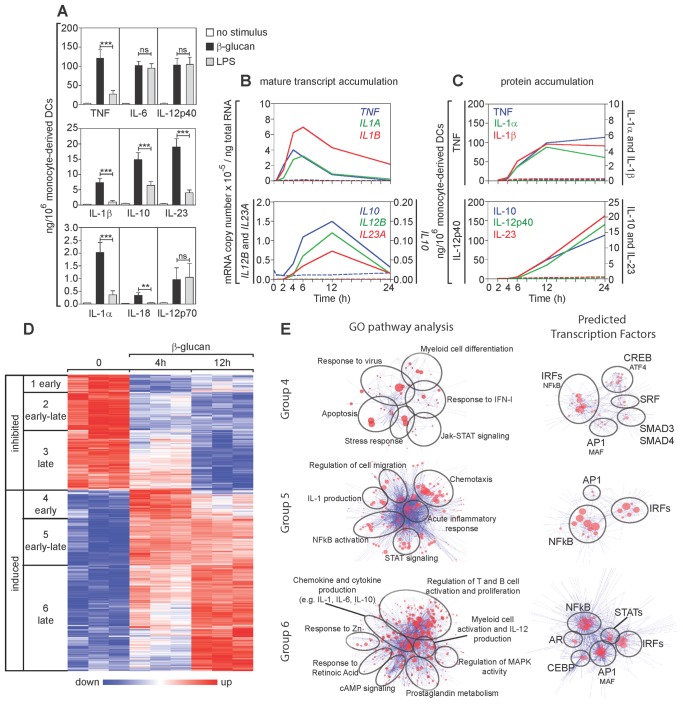
Gene expression in β-glucan-stimulated human DCs. (A) Human monocyte-derived DCs were cultured for 24 h with or without particulate β-glucan or LPS. Protein secretion was measured by ELISA in culture supernatants. Results are mean ± SEM, n = 12 to 31. Statistics: Wilcoxon signed-rank test (TNF, IL-6, IL-12p40, and IL-10) or Tobit model analysis (IL-1β, IL-23, IL-1α, IL-18, and IL-12p70). (B and C) Human monocyte-derived DCs were cultured in the presence (solid lines) or absence (dashed lines) of particulate β-glucan for the times shown. Transcript accumulation was determined by absolute Real-Time qRT-PCR using total RNA and primers detecting mature transcripts (Table S2 in File S1). Protein secretion was measured by ELISA in the culture supernatants. Results are mean, n = 6 to 8 for transcripts, n = 5 to 13 for proteins. (D) Gene expression in human monocyte-derived DCs was evaluated by microarrays after activation with β-glucan for the indicated times. The heat map shows differentially expressed [≥2-fold change in expression (FDR <0.1) in at least one time point compared to the pre-stimulation level] genes (rows) in cells from three different donors (columns). The mRNA profiles were hierarchically clustered and subdivided into six groups (leftmost column of each heat map) according to the time when β-glucan controlled their expression. (E) Cluster models (Cluster ONE Cytoscape plugin) illustrating the pathways associated with the 3 groups of genes induced by β-glucan (GO pathway analysis) (left panels) and their predicted TFs (TRANSFAC database) (right panels). Each node of the pathway clusters depicts a single pathway. Similar pathways were grouped under a single representative name. Each node of the TF clusters indicates a redundant name or a predicted binding site for the listed TFs. The size of the nodes indicates the significance of the association of the pathway or the potential binding site for a TF.

Although both TLR-agonists and ligands for lectin-type receptors are potent activators of human DCs, the two types of stimuli induce a different pattern of cytokine secretion [26]. In this study we analyzed the mechanisms by which β-glucan, a ligand for the Dectin-1 receptor, regulates activation and cytokine production in human monocyte-derived DCs using as a comparison LPS, the prototype TLR4-agonist. Confirming and extending our previous results [26], β-glucan induced human DCs to release higher levels of TNF, IL-10, IL-23, and IL-1 as compared to LPS, while IL-6, IL-12p40, and IL-12p70 were produced in similar amounts in response to either stimulus (Figure 1A).

As described for TLR-induced gene expression [38], the kinetics of mRNA accumulation and protein secretion of the cytokines produced by β-glucan-activated DCs also allowed us to classify their genes on the basis of their early or late expression following stimulation. Accumulation of mRNA of the early genes *TNF* and *IL1* was maximal between 4 and 6 h after activation, while protein was detectable at 4 h and reached a plateau at 12 h (Figures 1B and 1C). In contrast, mRNA of the late genes *IL10*, *IL12B*, and *IL23A* reached maximal accumulation only at 12 h and protein was not detectable until 6 h, increasing until at least 24 h. Microarray analysis showed that approximately 1500 genes were either inhibited (38%) or induced (62%) after stimulation with β-glucan (Figure 1D). Based on expression at 4 and/or 12 h, times that were identified in the preliminary PCR analysis to be optimal for detection of expression of early and late genes, the differentially regulated genes were divided into 6 groups: 1) early-inhibited genes; 2) genes inhibited similarly at both early and late times (early-late-inhibited genes); 3) late-inhibited genes; 4) early-induced genes; 5) genes induced similarly at both early and late times (early-late-induced genes); 6) late-induced genes (Figure 1D).

We focused only on β-glucan-induced genes, which included those encoding DC activation markers and immunoregulatory cytokines. These genes were analyzed for gene ontology (GO) pathways and for predicted regulation by TFs (Figure 1E). Early-induced genes (group 4) were those characteristic for apoptosis or stress, IFN-I and antiviral responses, and were predicted to be regulated mainly by interferon regulatory factors (IRFs). The early-late genes (group 5) were associated with chemotaxis, IL-1 production, and NF-κB or STAT signaling and were predicted to be activated mainly by NF-κB and IRFs. Finally, the late-induced genes (group 6) were those encoding chemokines and cytokines as well as factors involved in activation and proliferation of immune cells. These late genes were also predicted to be controlled by NF-κB and IRFs, but with a contribution of AP1, STATs, and CEBPs.

### Endogenous IL-1 controls β-glucan-associated gene expression in human DCs

**Figure 2:**
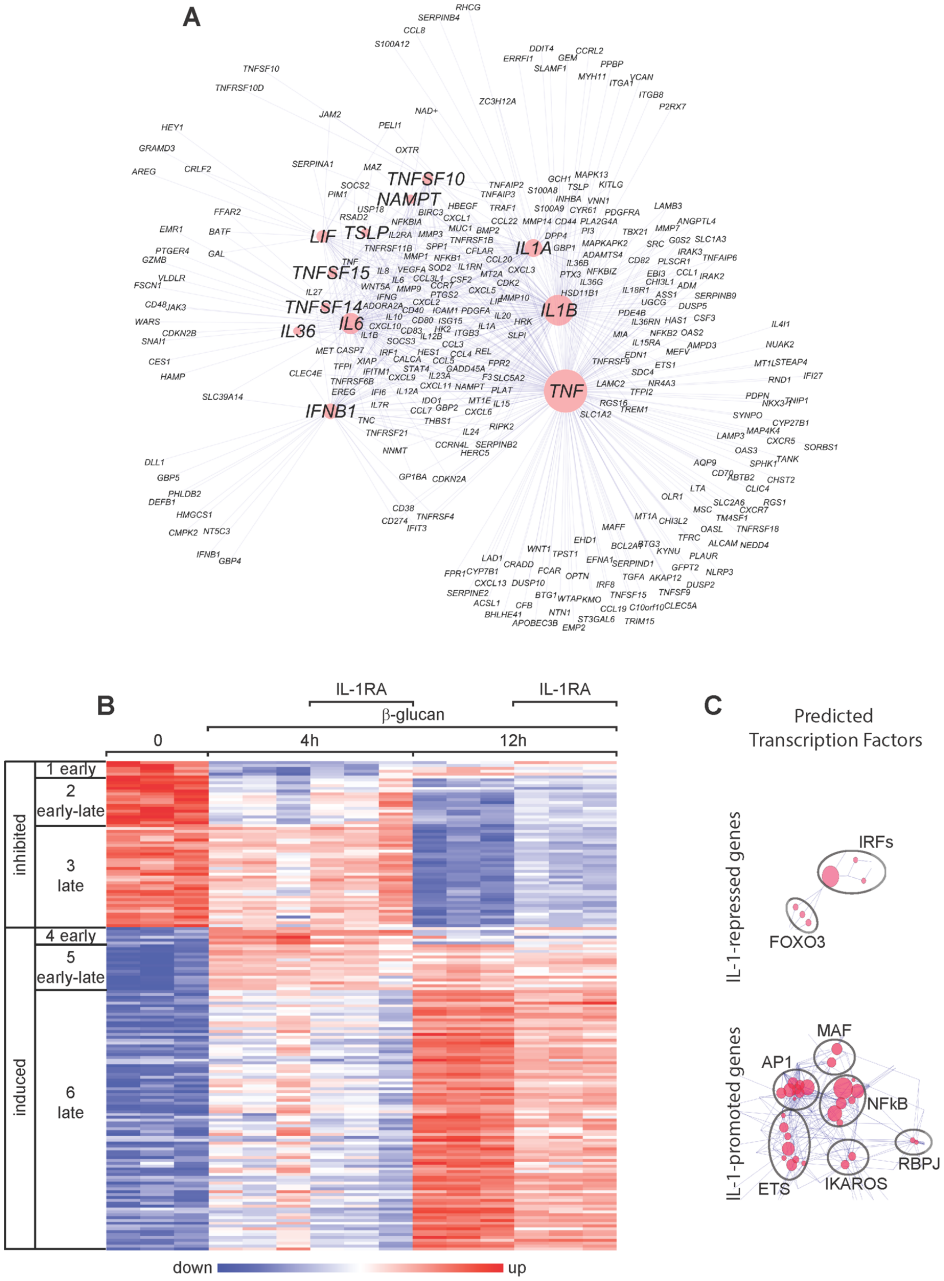
Gene expression in β-glucan-stimulated human DCs and its modulation by IL-1RA. (A) The gene expression data in Figure 1D were analyzed using the Ingenuity data set. The list of genes from groups 4 and 5 in Figure 1D was used to identify the potential regulators, whereas the genes in groups 5 and 6 were examined as potential targets. The cluster model shown (Cluster ONE Cytoscape plugin) illustrates predicted regulators (marked symbols in red circles) of the gene signature (small symbols) induced by β-glucan in monocyte-derived DCs. The dimensions of the red circles are proportionate to the predicted impact of the candidate regulators in the response to β-glucan. (B) Gene expression (microarrays) in human monocyte-derived DCs activated with β-glucan with or without IL-1RA (2.5 µg/ml) for the indicated times. The heat map includes the mRNA profiles of differentially expressed [≥2-fold change in expression (FDR<0.1) in the presence or absence of IL-1RA] genes (rows) in cells from three different donors (columns). The mRNA profiles were hierarchically clustered and then subdivided into six groups (leftmost column) according to the time when β-glucan controls their expression. (C) Cluster models with predicted TFs for the genes in groups 4, 5, and 6 repressed or promoted by IL-1.

The Ingenuity data set applied to the genome-wide gene expression data in Figure 1D uncovered several potential regulators of the β-glucan-induced gene expression in human DCs. These included the early-induced cytokines IL-1, TNF and IFN-I, and the early-late-induced cytokine IL-6 (Figure 2A).

We focused on the effects of the early-induced cytokines, initially analyzing endogenous IL-1. We determined genome-wide gene expression in response to β-glucan and its regulation by IL-1 receptor antagonist (IL-1RA) in human monocyte-derived DCs activated for 4 and 12 h. Perturbation of IL-1 signaling by IL-1RA modified the expression of 20% of β-glucan-regulated genes with the most evident effect on the early-late and late genes expressed at 12 h (Figure 2B).

Among the β-glucan-induced genes, only a few were repressed by IL-1. These IL-1-repressed genes were those characteristic of the IFN-I response and were predicted to be regulated mainly by IRFs. Instead, the genes promoted by the positive feedback of IL-1 were mostly those encoding cytokines and factors involved in T cell activation and proliferation and were predicted to be regulated mainly by NF-κB, AP1, and ETS (Figure 2C).

### TNF and IFN-I are shared modulators of DC activation, whereas IL-1 selectively supports the β-glucan response

**Figure 3:**
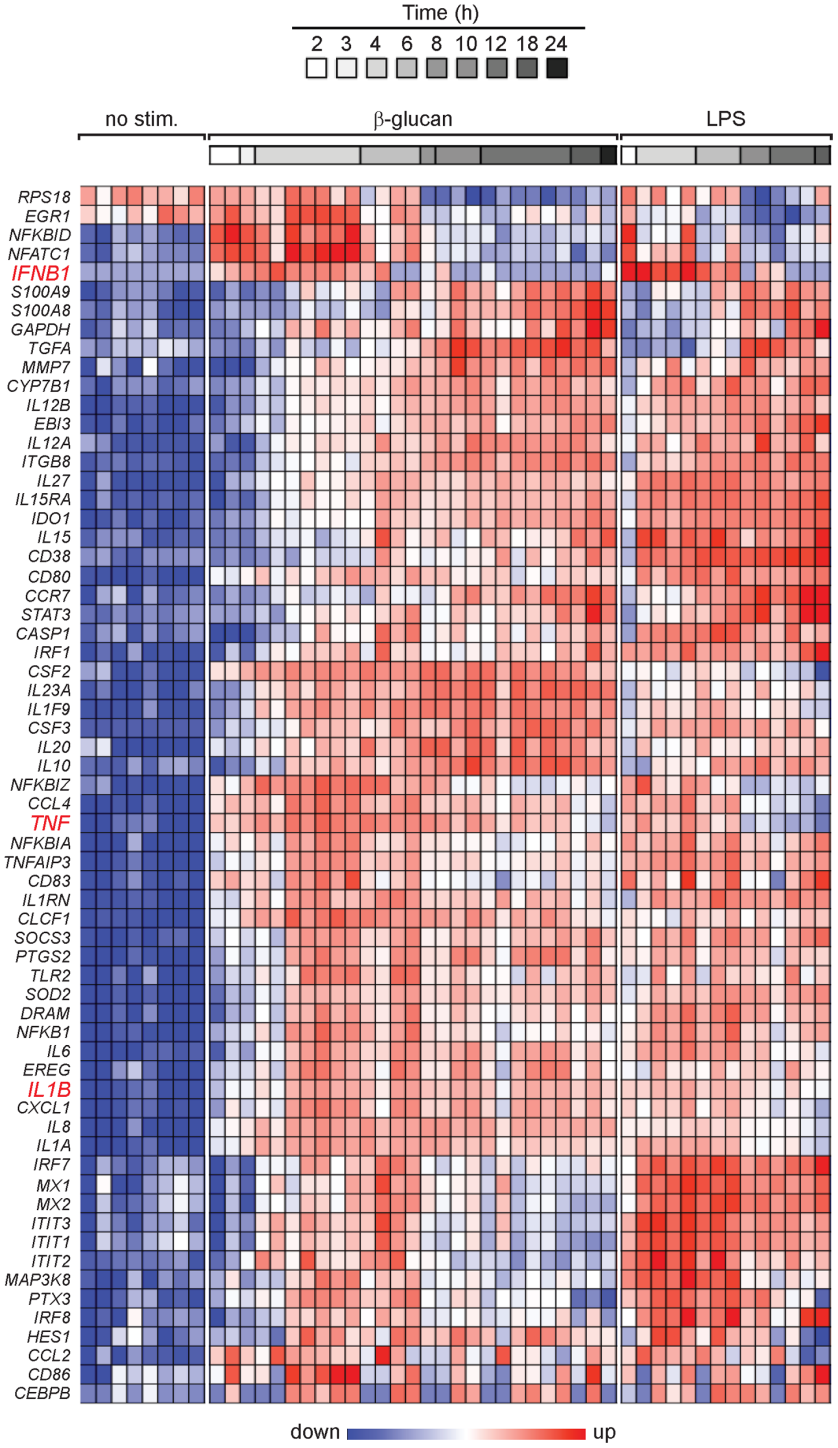
Expression profiles of immunoregulatory genes in DCs activated by β-glucan or LPS. Human monocyte-derived DCs were cultured in the presence or absence of particulate β-glucan or LPS for the times shown. mRNA levels in cell lysates for 64 selected genes were quantitated by NanoString's nCounter technology. The gene symbols are listed in the leftmost column of the heat map. Technical replicates were collapsed and genes hierarchically clustered (rows, genes; columns, donors). Data from individual donors are expressed as fold change between the mRNA counts of each condition and the corresponding average levels of mRNA (baseline) across donors, times, and treatments.

Sixty-four β-glucan-induced genes, encoding transcriptional and immunoregulatory factors predicted to be relevant for DC functions and inflammation, were selected on the basis of the microarray data for further gene expression analysis using the NanoString's nCounter system (Table S1 in File S1). Both IL-1-dependent and -independent genes were among those selected. Gene expression kinetics were analyzed in human monocyte-derived DCs stimulated by either β-glucan or the TLR4-agonist LPS as a comparison to a prototypical TLR stimulation (Figure 3). Transcripts for the genes encoding proIL-1β, TNF, and IFN-β as well as the NF-κB family TFs (e.g., *NFKBIZ* and *NFKBID*) accumulated early following stimulation with either ligand (Figure 3). However, the expression of *IFNB1* and genes encoding families of IFN-associated mediators and TFs (*IFIT*, *IRF*, and *MX*) was stronger and longer lasting with LPS than with β-glucan. Late genes encoding certain leukocyte activation markers and antiviral response molecules (e.g., *IL15*, *CD38*, *CD80*, *CCR7*, and *IRF1*) were preferentially activated by LPS, whereas those encoding certain immunoregulatory cytokines (e.g., *IL1F9*, *IL10*, *IL20*, *IL23A*, *CSF2*, and *CSF3*) showed greater induction by β-glucan.

**Figure 4:**
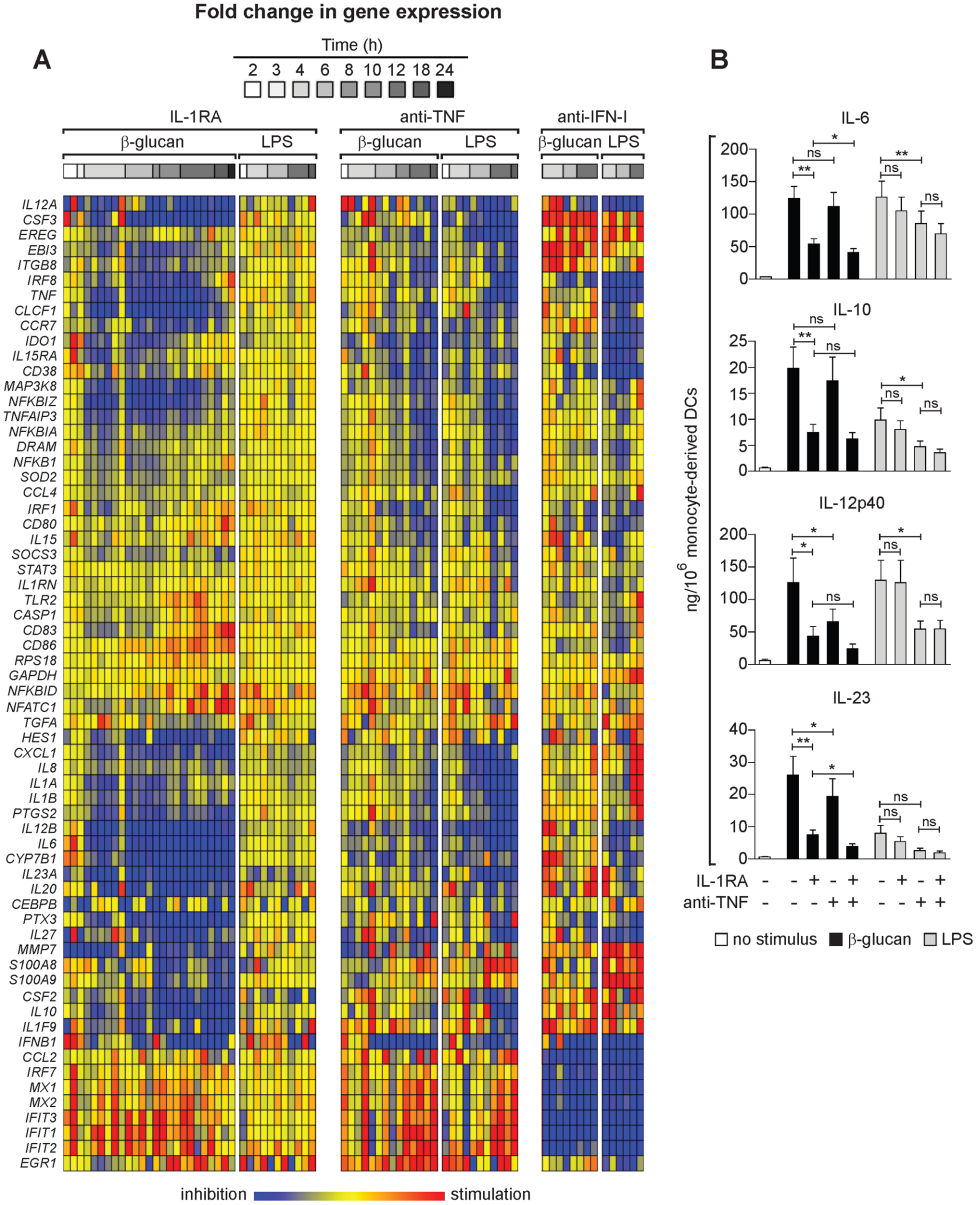
Regulation of the DC response to β-glucan or LPS by IL-1, TNF, and IFN-I. (A) Human monocytes-derived DCs were stimulated with particulate β-glucan or LPS, in the presence or absence of IL-1RA (25 µg/ml) or the indicated neutralizing antibodies, for the times shown. mRNA levels in cell lysates for 64 selected genes were quantitated by NanoString's nCounter technology. The gene symbols are listed in the leftmost column of the heat map. Technical replicates were collapsed and genes hierarchically clustered (rows, genes; columns, donors). Results from individual donors were analyzed as fold change between the mRNA counts of each condition and the corresponding average levels of mRNA (baseline) across donors, times, and treatments. Final data are expressed as fold change between the mRNA counts of each condition and the corresponding baseline mRNA counts from the same cells in the absence of IL-1RA or antibodies (red, stimulation from baseline; blue, inhibition from baseline; yellow, unchanged from baseline). The heat map reports the results with all the genes analyzed by NanoString technology. The data for all the comparisons were analyzed by one-way ANOVA model using Methods of Moments [65]. The modulated genes mentioned in the text are those in which the changes induced by the perturbations were significant (p<0.05) at least at 12 h post-stimulation. (B) Human monocyte-derived DCs were cultured for 24 h as in (A). Protein secretion in the culture supernatants was measured by ELISA. Results are mean ± SEM, n = 5 to 10. Statistics: paired t-test when measurements were uncensored or the Tobit model analysis in presence of left-censored data.

To further understand the effect of the endogenous early cytokines IL1, TNF, and IFN-I on gene induction in activated DCs, we studied the perturbation of the cytokine signaling by IL-1RA, and antibodies neutralizing TNF and IFN-I (Figure 4A). Unlike β-glucan, and consistent with published data [39], LPS induced in human DCs a negligible release of IL-1α and IL-1β (Figure 1A). IL-1RA selectively modulated the response of human monocyte-derived DCs to β-glucan and had no unspecific effects on the LPS response (Figure 4 and Figure S1 in File S1). IL-1 inhibition down-modulated the expression of genes encoding immunoregulatory and Th-inducing cytokines, antifungal and antibacterial molecules as well as cell adhesion and migration mediators (e.g., *IL6*, *CLCF1*, *IL12A*, *IL12B*, *IL27*, *IL1F9*, *IL10*, *IL20*, *IL23A*, *CSF2*, *CSF3*, *PTX3*, *MMP7*, *CXCL1*, *CCR7*, and *CYP7B1*). By contrast, IL-1 antagonism did not affect or only marginally affected several regulators of inflammation, co-stimulatory molecules and other genes controlling cell adhesion, migration or proliferation (e.g., *NFKBID*, *SOCS3*, *STAT3*, *DRAM*, *CEBPB*, *ITGB8*, *CCL2*, *CCL4*, *EREG*, *IDO1*, *CD80*, *CD83*, and *CD86*). Interestingly, IL-1RA also inhibited expression of *TNF* and of the TFs *NFKBIZ* and *IRF8*. *IFNB1* expression was up-regulated in DCs treated with IL-1RA within the first 3 h of culture but quickly down-modulated, although the expression of the IFN-regulated genes *IFIT1*, *IFIT2*, and *IFIT3* was consistently up-regulated.

TNF neutralization reduced the expression of immunoregulatory genes and promoted the accumulation of IFN-associated transcripts in both β-glucan- and LPS-stimulated DCs (Figure 4 and Figure S1 in File S1). The genes regulated by IL-1 in β-glucan-stimulated DCs and by TNF in both β-glucan- and LPS-exposed DCs largely overlapped, although the effect of TNF neutralization on β-glucan-induced gene expression was observed later than that of IL-1RA (Figure 4A). These results suggest that the IL-1 feedback is required for the full activation of DCs by β-glucan, while the TNF feedback mostly acts by prolonging the length of the DC response.

IFN-I neutralization in both β-glucan- and LPS-exposed DCs down-modulated all the antiviral response genes as well as *IL27*, *IL12A*, *CCL2*, and *CD38*, while it promoted the expression of genes encoding several immunoregulatory cytokines and molecules involved in cell adhesion and proliferation (Figure 4A). Noteworthy, among the genes dependent on IFN-I for optimal expression, it was possible to distinguish those that were unaffected or up-regulated by IL-1RA and anti-TNF antibody, including *CCL2, IRF7, MX1/2, IFT1,2,3* and others that also required TNF (and for most of them IL-1 in β-glucan-activated DCs) for optimal expression, including *IL12A, IRF8, ITGB8, TNF, CCR7, IDO1, IL-15RA, CD38*, and *IFNB1*. In LPS-simulated DCs, IFN-I neutralization up-regulated the late expression of *IL1A, IL1B, IL8*, and *CXCL1*. IFN-I blockade did not alter the expression of NF-κB family TFs, including *NFKBIZ*, nor the expression of *STAT3* and *CEBPB* in response to either β-glucan or LPS (Figure 4A).

Overall, these data show that: 1) *IL1*, *TNF*, and *IFNB1* gene expression is induced in human DCs by both β-glucan and LPS, although IL-1 protein is only released in the presence of the first stimulus; 2) TNF and IFN-I are shared modulators of DC activation by β-glucan and LPS; 3) IL-1 and IFN-I antagonize each other in β-glucan-stimulated DCs and are required for DC early activation; 4) TNF mostly acts by prolonging the β-glucan- or LPS-induced response, while it antagonizes the expression of IFN-I-dependent genes; 5) The distinctive feedback regulation of gene expression in β-glucan-activated DCs endows these APCs with an IL-1-dependent signature of immunomodulatory genes that could be predicted to affect the regulation of the class of immune response.

### Endogenous IL-1 promotes β-glucan induction of late immunoregulatory cytokines at the transcriptional level

**Figure 5:**
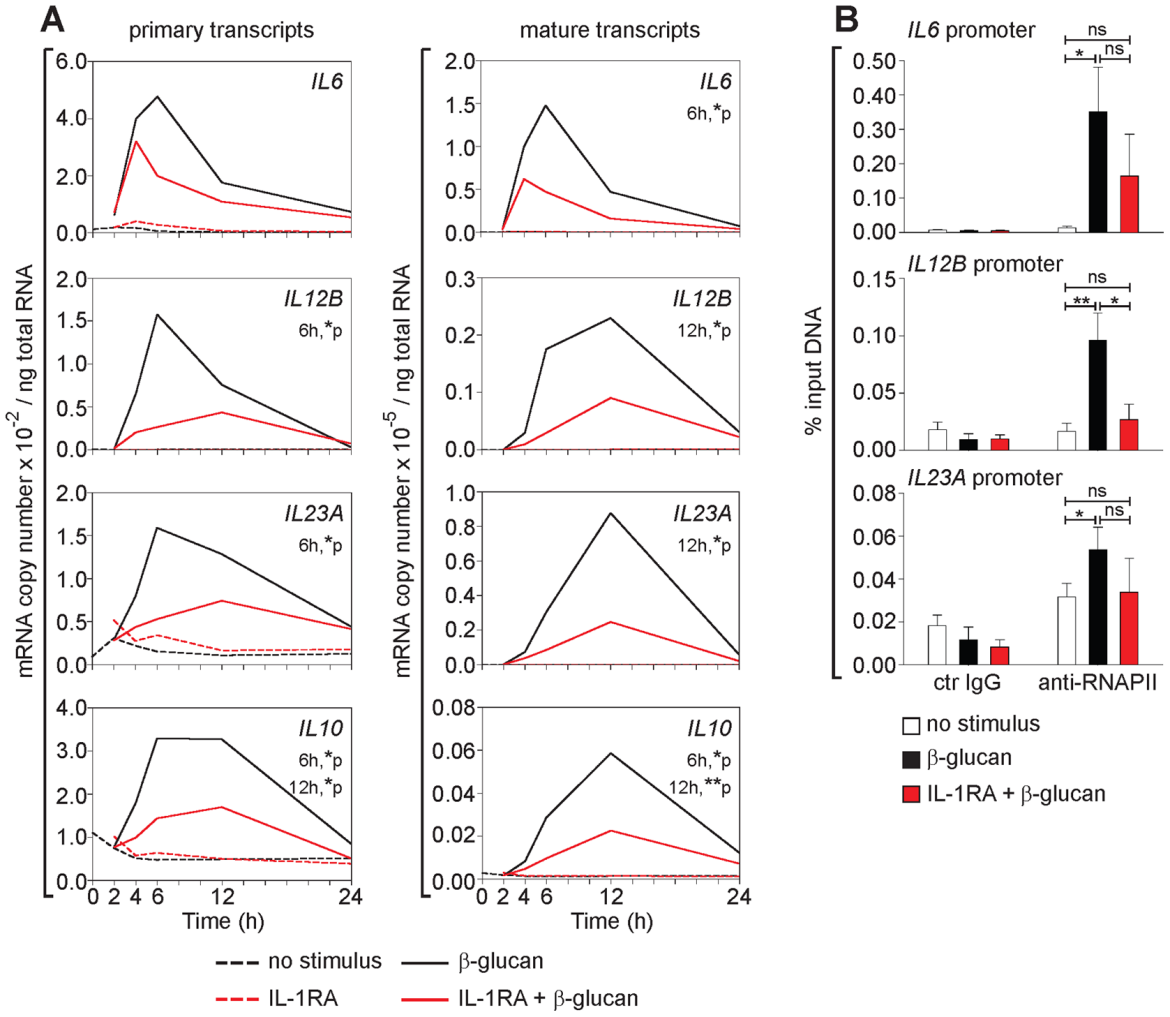
IL-1-mediated transcriptional regulation of β-glucan-induced late immunoregulatory cytokines in human DCs. (A) Human monocyte-derived DCs were cultured with or without particulate β-glucan and IL-1RA (2.5 µg/ml). Transcript accumulation was determined by absolute Real-Time qRT-PCR using total RNA and primers (Table S2 in File S1) for the detection of either primary or mature transcripts. Results are mean, n = 4. The times when significant differences were found as well as their p values are indicated in the figure. (B) Human monocyte-derived DCs were cultured in the presence or absence of particulate β-glucan and IL-1RA (25 µg/ml) for 6 h. ChIP was performed using sheared chromatin from fixed cells. DNA-protein complexes were immunoprecipitated with control IgG or RNAPII antibody (anti-RNAPII). Eluted DNA was analyzed by Real-Time qRT-PCR using primers (Table S2 in File S1) specific for *IL6*, *IL12B*, and *IL23A* promoter regions, proximal to the transcription start site. Results are mean ± SEM, n = 4. Statistics: paired t-test.

We next analyzed the molecular mechanisms by which the MyD88-dependent signaling by endogenous IL-1 regulates gene expression in β-glucan-stimulated DCs. The kinetics of induction of primary (unspliced) transcripts and of accumulation of mature mRNA for late cytokine genes, after perturbation of IL-1 signaling by IL-1RA, were analyzed in activated human monocyte-derived DCs. The primary transcript levels were 2-3 orders of magnitude lower than those of mature transcripts and their peak of induction preceded that of accumulation of mature transcripts (Figure 5A). IL-1RA significantly decreased both β-glucan-induced primary and mature transcript accumulation of *IL6*, *IL12B*, *IL23A*, and *IL10* (Figure 5A) as well as the binding of RNA polymerase II (RNAPII) to the promoter of *IL12B* (Figure 5B), with no significant changes in mRNA stability (Figure S2 in File S1). These data indicate that in activated DCs IL-1 controls the β-glucan-induction of late cytokine genes at the transcriptional level. IL-1β but not IL-1α neutralizing antibodies mimicked the effect of IL-1RA (Figure S3A in File S1). Consistent with published data in murine phagocytic cells [29], [30], using specific inhibitors, we confirmed that β-glucan induced IL-1β in human DCs in a NLRP3 inflammasome- and caspase-1- and -8-dependent manner (data not shown and Figure S3B in File S1). Specific inhibition of caspase-8 and/or -1 also decreased the β-glucan induction of IL-10, IL-23, and IL-12p40 (Figure S3B in File S1). These results suggest that late cytokine production by β-glucan-activated DCs is regulated primarily by inflammasome-processed IL-1β. Because our data in Figure 1C show that IL-1α and IL-1β proteins are secreted at similar levels and with similar kinetics by β-glucan-stimulated human DCs, the apparently predominant role of IL-1β could be surprising. However: 1) unlike IL-1β, IL-1α is processed after secretion although the processing might not affect some of its biological activities [40]; 2) IL-1α secretion requires the inflammasome but in a capase-1-independent mechanism [41]: 3) IL-1α and IL-1β act on the same receptors and are expected to have similar activities, but differential effects of the two cytokines have been identified in other biological systems and attributed to different processing or cellular localization [42]. Thus, although β-glucan direct signaling appears to induce only an early gene expression profile in human DCs, its ability to concurrently activate the inflammasome recruits the IL-1-mediated feedback that allows the optimal expression of late genes and endows DCs with distinct immunoregulatory activities.

### Endogenous IL-1 supports the DC response to β-glucan by maintaining nuclear IκB-ζ for the modulation of immunoregulatory genes

**Figure 6:**
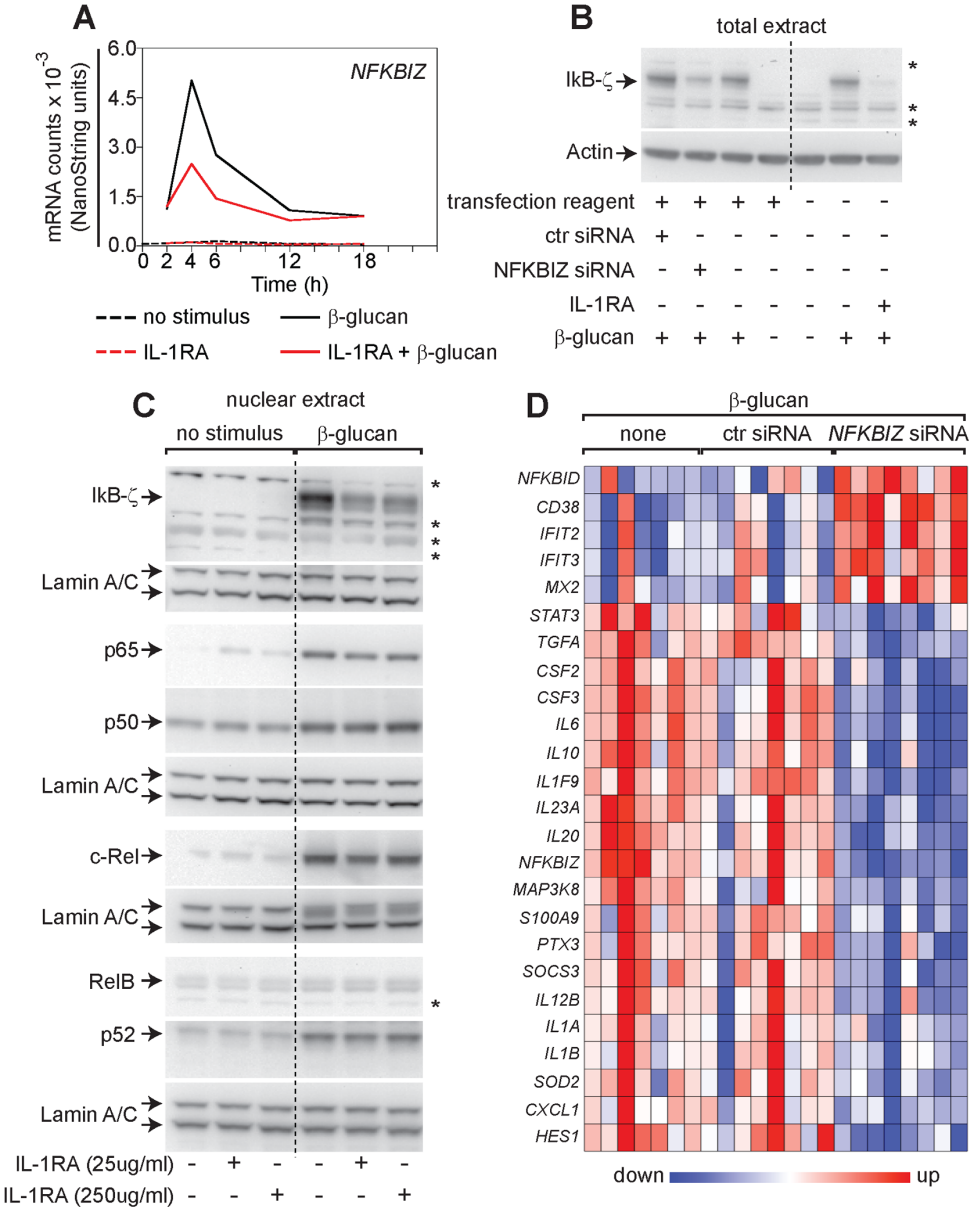
Regulation of the DC response to β-glucan by the IL-1-induced nuclear factor IκB-ζ. (A) Human monocyte-derived DCs were cultured with or without particulate β-glucan and/or IL-1RA (25 µg/ml). Gene expression data are from the experiment of Figure 4A and results shown are normalized mRNA counts from technical triplicates from one representative donor out of 8 analyzed. (B) Human monocyte-derived DCs were cultured for 12 h in the indicated condition. IL-1RA was used at 250 µg/ml. Total extracts were analyzed by Western blotting with the indicated antibodies. Data shown are from one representative donor out of 9 tested. Arrows indicate specific bands, while asterisks show nonspecific bands. (C) Human monocyte-derived DCs were cultured in the presence or absence of particulate β-glucan and/or IL-1RA as indicated for 6 h and nuclear extracts prepared for Western blotting. Data are from one donor and are representative of those obtained in at least 3 independent experiments with cells from different donors. Arrows indicate specific bands, while asterisks nonspecific bands. (D) Human monocyte-derived DCs from different donors were stimulated for 12 h with β-glucan in presence of transfection reagent (none) after pre-incubation with a non-targeting siRNA pool (ctr siRNA) or a pool of four siRNA directed against *NFKBIZ* (*NFBIZ* siRNA). mRNA levels in cell lysates for 64 selected genes were quantitated by NanoString's nCounter technology and the map shows genes with significant changes in expression (FDR<0.1) following knockdown of *NFKBIZ*. Results are expressed as in Figure 3.

Analysis of the expression kinetics and regulation by IL-1 of the β-glucan-induced TFs identified by the genome-wide gene expression study uncovered the *NFKBIZ* gene (encoding IκB-ζ) as the earliest expressed, β-glucan-induced, TF that was inhibited by IL-1RA. IL-1 antagonism reduced *NFKBIZ* mRNA accumulation and both total and nuclear IκB-ζ protein in human monocyte-derived DCs exposed to β-glucan, but had no effect on the nuclear localization of the canonical and non-canonical NF-κB subunits (Figures 6A, 6B, and 6C).

To test the involvement of IκB-ζ in the IL-1-mediated control of cytokine production induced by β-glucan, siRNA was used to knockdown *NFKBIZ* in activated human monocyte-derived DCs. Knockdown efficiency was evaluated by Western blotting (Figure 6B). Similar to IL-1 antagonism, *NFKBIZ* silencing down-modulated gene expression of cytokines and pro-inflammatory molecules, while it promoted expression of IFN-inducible genes (Figures 4A and 6D). Thus, the effect of *NFKBIZ* silencing closely resembled that of the IL-1 antagonism, suggesting that IκB-ζ is a key factor by which autocrine IL-1 regulates the response of human DCs to β-glucan.

### IκB-ζ enhances the human *IL23A* promoter activity induced by IL-1

**Figure 7:**
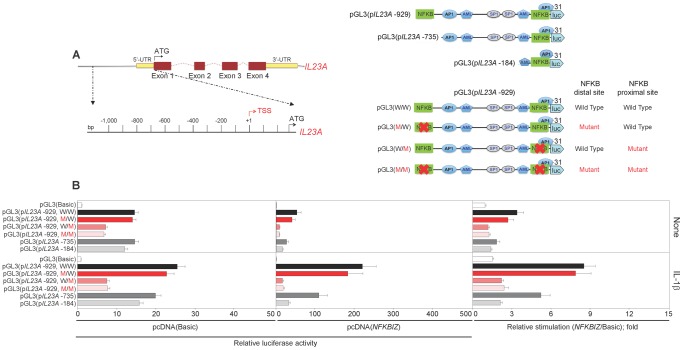
Regulation of the IL-1-induced activity of the human *IL23A* promoter by IκB-ζ. (A) Schematic representation of the human *IL23A* gene and strategy for the preparation of pGL3 luciferase reporter vectors under the control of different lengths of wild type or mutated *IL23A* 5′ upstream promoter sequences. The major predicted binding sites for TFs are depicted. (B) HeLa cells were transfected with a basic pcDNA3.1 plasmid, pcDNA(*Basic*), or a pcDNA(*NFKBIZ*) along with pGL3 luciferase reporter vectors under the control of different lengths of wild type or NF-κB-mutant promoter of *IL23A*. At 24 h after transfection, cells were treated with IL-1β. Luciferase activity was read in cell lysates after 48 h of stimulation. Data were normalized to TK-*Renilla* activity and expressed relative to the normalized activity of the Basic vector or as mean fold change ± SEM in normalized luciferase activity induced by the pcDNA(*NFKBIZ*) with respect to the control vector pcDNA(Basic) to highlight the *NFKBIZ*-stimulated luciferase activity from each reporter vector shown. Results are mean ± SEM, n = 6.

Our present data indicate that β-glucan-induced IL-6 and IL-23, two cytokines with important immunoregulatory functions on the Th cell responses, are promoted at the transcriptional level by the IL-1/IκB-ζ axis and we previously reported that IL-1β and IκB-ζ induce human *IL6* promoter activity [34]. Here, we analyzed whether IκB-ζ has a direct effect on the human *IL23A* promoter. The 1000 bp sequence upstream of the transcription start site (TSS) of human *IL23A* contains two putative NF-κB binding sites at positions -884 and -90 and one putative AP-1 site at position -673. pGL3 luciferase reporter vectors under the control of different lengths of wild type *IL23A* 5′ upstream promoter (-929, containing both NF-κB site; -735 and -184 containing only the proximal NF-κB site) were tested (Figure 7A). The -929 and, to a lesser extent, the -735 and the -184 constructs were induced in HeLa cells by IL-1β. Mutation of either the proximal or both NF-κB sites almost extinguished the responsiveness of the -929 construct, whereas mutation of the distal site had no effect (Figure 7B). Overexpression of *NFKBIZ* strongly potentiated the IL-1β-dependent induction of the -735 and -929 constructs (provided that the proximal NF-κB site was intact), but had only a minor effect on unstimulated HeLa cells or on the basic pGL3 vector (Figure 7B). Although the -735 and the -184 constructs were induced at similar levels by IL-1β, overexpression of *NFKBIZ* enhanced approximately six-fold the induction of the -735 construct and only two-fold that of the -184 construct, comparable with the induction seen with the basic pGL3 vector (Figure 7B). A -665 construct deleting the AP-1 site behaved similarly to the -735 construct (Figure S4 in File S1). Overall, these data indicate that the -90 NF-κB site is essential for *IL23A* promoter activity in response to IL-1β with enhancer elements present between -184 and -665. IκB-ζ affects the *IL23A* promoter by potentiating the response to IL-1β acting on the enhancer elements and has less or no activity on the proximal NF-κB element.

### IFN-γ reprograms the ability of β-glucan-activated DCs to induce Th cell polarization by mimicking the effect of IL-1 antagonism

**Figure 8:**
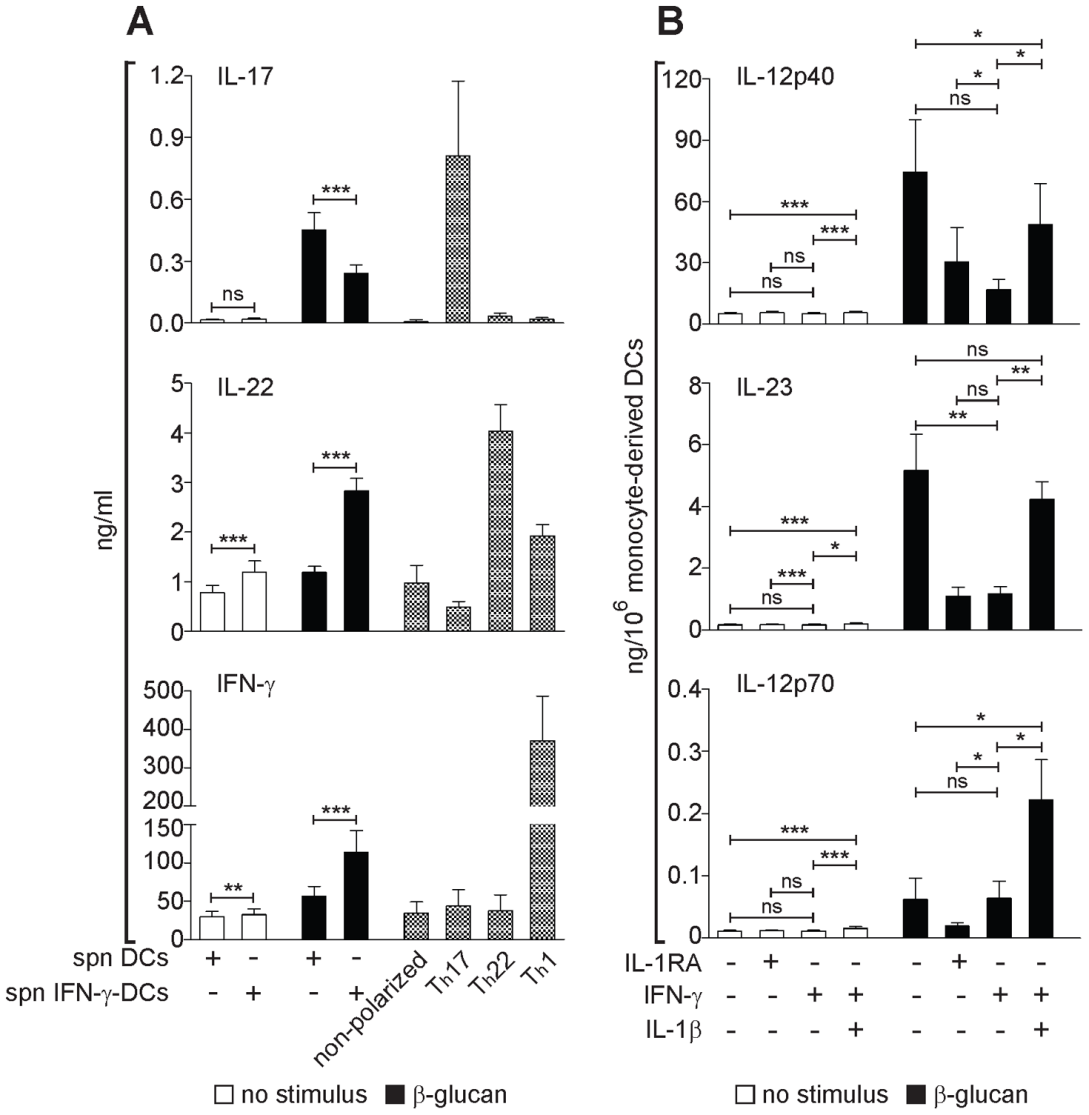
Regulation of DC-promoted Th cell responses to β-glucan by IFN-γ. (A) Human monocyte-derived DCs, unprimed (DCs) or primed overnight with IFN-γ (IFN-γ-DCs), were washed extensively and cultured in the presence or absence of β-glucan for 24 h. Culture supernatants (spn) were used to polarize freshly isolated naïve CD45RO^-^CD4^+^ T lymphocytes in presence of anti-CD3 and anti-CD28 antibodies. After 4 d, T cells were washed and stimulated with anti-CD3 and PMA for further 18 h. As controls, naïve T cells were also differentiated with optimal combinations of immunoregulatory cytokines into Th17, Th22, and Th1 cells as described in Materials and Methods S1 in File S2. Cytokine secretion was measured by ELISA. Results are mean ± SEM using monocyte-derived DC supernatants from 4 donors, each tested on T cells from 4 different donors. Statistics: Wilcoxon signed-rank test. (B) Human monocyte-derived DCs, unprimed or primed overnight with IFN-γ were stimulated or not for 24 h with β-glucan, IL-1RA (250 µg/ml) and/or IL-1β as indicated. Protein secretion in the culture supernatants was measured by ELISA. Results are mean ± SEM, n = 6 to 9. Statistics: paired t-test when measurements were uncensored or Tobit model analysis in presence of left-censored data.

In addition to autocrine/paracrine mechanisms, the activation of APCs is regulated in the inflammatory environment by cytokines produced by innate cells, such as other inflammatory cells or innate lymphocytes, as well as by immune lymphocytes. In particular IFN-γ produced by NK cells or by activated T cells has a major modulatory effect on DC differentiation/activation and on the production of IL-12 family cytokines that orchestrate the Th1/Th17 immune response [10], [26]. We reported that IFN-γ priming of DCs inhibits the β-glucan-induction of the Th17-promoting cytokine IL-23, while it increases the Th1-polarizing cytokine IL-12p70 [26], but the exact mechanism of this innate cytokine modulation and its impact on the adaptive immune response to β-glucan remained unclear. To assess the role of IFN-γ in modulating DC programming by β-glucan for Th cell polarization, we cultured naïve CD4^+^ T lymphocytes with plate-bound anti-CD3 and conditioned media from monocyte-derived DCs activated by β-glucan in presence or not of IFN-γ (Figure 8A). Consistent with our previous findings [26], conditioned media from monocyte-derived DCs stimulated with β-glucan induced IL-17-producing Th cells resembling, for cytokine release, the Th17-like cells differentiated with optimal doses of IL-1β, IL-6, and IL-23 (Th17 polarization, Figure 8A). IFN-γ priming of monocyte-derived DCs halved the production of IL-17 by polarized T cells in response to β-glucan, but doubled the release of Th-derived IFN-γ and IL-22 (Figure 8A).

IFN-γ priming of β-glucan-activated monocyte-derived DCs mimicked the effect of IL-1 inhibition by IL-1RA and inhibited the secretion of IL-12p40 and IL-23, but not IL-12p70. Recombinant IL-1β completely restored the levels of IL-23 that were repressed by IFN-γ (Figure 8B).

Overall these data show that IFN-γ reprograms β-glucan-stimulated human DCs to promote the differentiation of T cells producing IFN-γ/IL-22 rather than IL-17. This effect seems to be mediated by the ability of IFN-γ to interfere with the endogenous IL-1-mediated positive feedback signaling.

### IFN-γ reprograms β-glucan-activated DCs by affecting both the production of IL-1β and its ability to induce IκB-ζ

**Figure 9:**
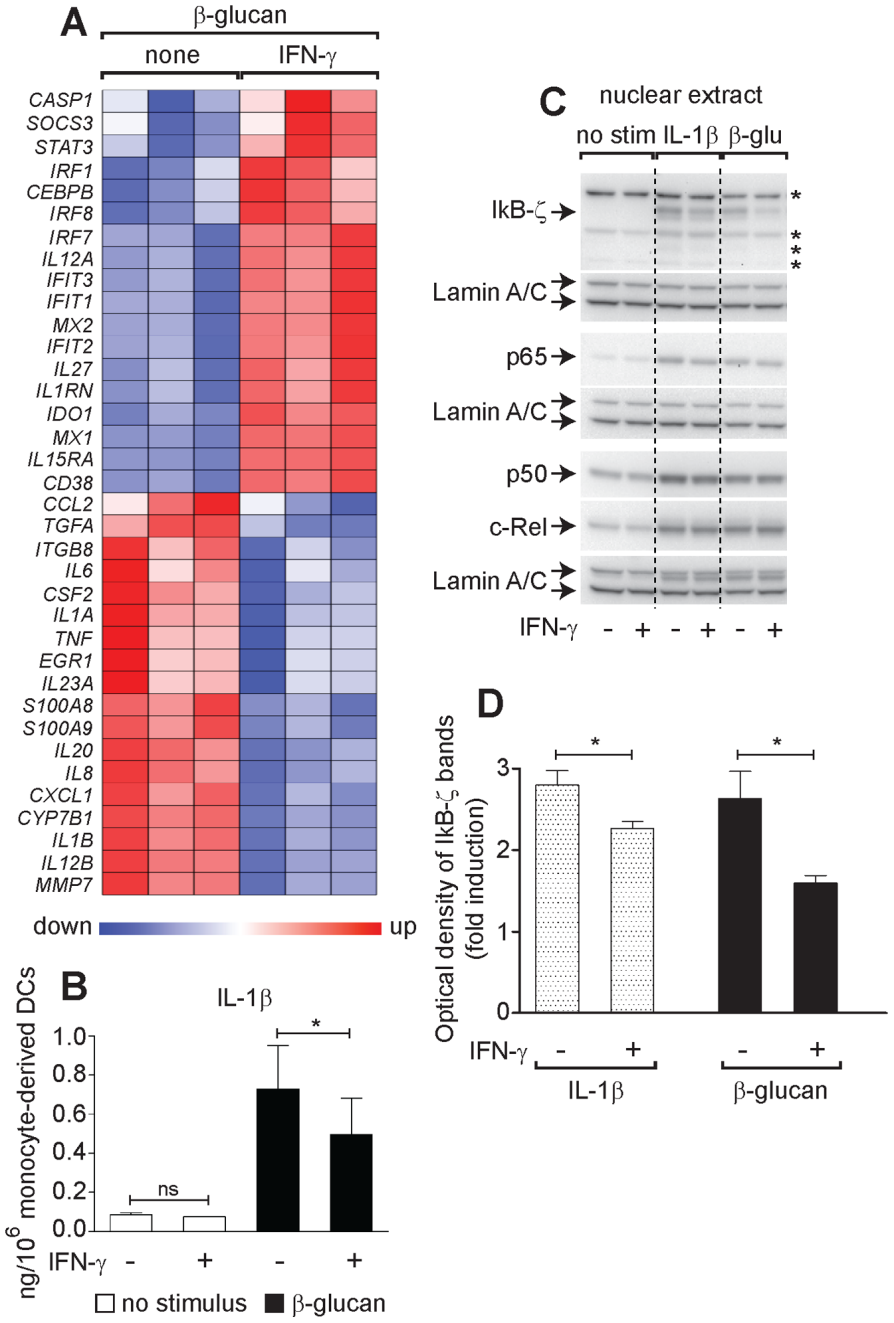
IFN-γ-mediated regulation of immune response to β-glucan *via* inhibition of IL-1β and IκB-ζ in activated DCs. (A) Human monocyte-derived DCs, unprimed or primed overnight with IFN-γ were stimulated or not for 12 h with β-glucan as indicated. mRNA levels in cell lysates for 64 selected genes were quantitated by NanoString's nCounter technology and the map shows genes with significant changes in expression (FDR<0.1) upon IFN-γ. Results from 3 different donors are expressed as in Figure 3. (B) Human monocyte-derived DCs, unprimed or primed overnight with IFN-γ were stimulated or not for 24 h with β-glucan as indicated. Protein secretion in the culture supernatants was measured by ELISA. Results are mean ± SEM, n = 7. Statistics: Tobit model analysis. (C) Human monocyte-derived DCs, unprimed or primed overnight with IFN-γ were cultured in the presence or not of particulate β-glucan as indicated for 6 h and nuclear extracts prepared for Western blotting. Data are from one donor and are representative of those obtained in at least 3 independent experiments with cells derived from different donors. Arrows indicate specific bands, while asterisks nonspecific bands. (D) Optical density of nuclear IκB-ζ bands from Western blotting analysis in (C). Data shown are fold induction over background from unstimulated cells. Results are mean ± SEM, n = 3. Statistics: t-test.

The observation that IFN-γ priming of β-glucan-activated DCs mimicked the effect of IL-1RA and was counteracted by exogenous IL-1β suggested that IFN-γ may affect the IL-1/IκB-ζ pathway underlying DC programming by β-glucan. Indeed, IFN-γ priming of monocyte-derived DCs reduced the expression of many of the β-glucan-induced genes that were also repressed by IL-1RA or *NFKBIZ* siRNA. IFN-γ priming up-regulated the IFN-responsive genes that were also induced by IL-1RA and *NFKBIZ* siRNA (e.g. *IFIT3*, *MX1*), but in addition it increased the expression of another group of IFN-responsive genes (*IRF8*, *SOCS3*, *STAT3*, *IL-12A*, *IL-27, CEBPB* and *IL1RN*) that were repressed or unaffected by IL-1RA (Figures 4A, 6D, and 9A). IL-1β secretion induced by β-glucan was decreased by IFN-γ by 50% at 12 h and 30% at 24 h (Figure 9B). IFN-γ priming of monocyte-derived DCs also inhibited the nuclear accumulation of IκB-ζ, but not the canonical NF-κB family members induced by a 6-h culture with β-glucan (Figures 9C and 9D). Recombinant IL-1β restored the levels of IκB-ζ that were repressed by IFN-γ in β-glucan-stimulated DCs. IFN-γ priming of DCs did not alter IL-1R1 and IL-1RAcP surface expression (Figure S5 in File S1), but slightly decreased IκB-ζ nuclear localization in response to the recombinant IL-1β (Figures 9C and 9D). Overall these data suggest that IFN-γ acts on β-glucan-activated DCs by negatively affecting both production of IL-1β and its ability to induce IκB-ζ.

## Discussion

Innate receptors regulate the transcriptional activation of DCs, thus modulating inflammation and adaptive immunity. TLR-regulated gene expression has been studied in great detail in the mouse [35], [43], but the molecular networks activated in APCs by innate receptors, other than TLRs, are less well characterized. We used a gene expression/perturbation approach to uncover the molecular basis of the activation of human DCs by β-glucan. β-glucan signaling through Dectin-1 programs DCs to produce cytokines such as IL-1, IL-6, and IL-23 that favor Th17 and, less efficiently, Th1 responses [44]. We now demonstrate that IL-1 and IFN-γ differentially regulate the β-glucan-induced Th responses by controlling the programming of stimulated human DCs *via* IκB-ζ modulation.

As known for TLR agonists [14], β-glucan regulated gene expression in human DCs with different kinetics. Genes such as *IL1, IFNB1*, *TNF*, and *NFKBIZ* were induced by β-glucan within a few hours after stimulation, whereas the transcription of other immunoregulatory genes including *IL6*, *IL10*, *IL12*, and *IL23*, crucial for Th cell differentiation and functional regulation, was activated at a later time. Unlike the control LPS, β-glucan induced human monocyte-derived DCs to release high levels of protein IL-1β through the activation of the NLRP3 inflammasome (data not shown), as previously reported in human macrophages [28]. The early cytokines IFN-I and TNF modulated in an autocrine manner the late transcriptional response to both β-glucan and LPS, while blockade of autocrine IL-1 selectively regulated the β-glucan-induced programming of human DCs.

β-glucan promoted in human DCs an IFN-I gene signature consistent with the reported induction of IFN-I in response to fungi *via* Dectin-1 signaling [45]-[47]. Preventing autocrine IFN-I signaling repressed antiviral response genes in DCs stimulated with either β-glucan or the control LPS. We previously showed a similar feedback effect of IFN-I in *M. tuberculosis*-infected human macrophages [17], while others recently identified the IFN-I pathway as crucial for host defense against *C. albicans* mediated by human peripheral blood mononuclear cells [47]. *C. albicans* and certain *Mycobacterium spp.* contain β-glucan or other Dectin-1 ligands [47], [48]. Thus, our data suggest that the biologically important IFN-I response induced by fungal pathogens and mycobacteria [47], [49] might be in part mediated by the β-glucan-dependent activation of Dectin-1.

Neutralization of autocrine TNF in DCs exposed either to β-glucan or LPS repressed the expression of NF-κB-regulated genes, while it promoted the IFN-I gene signature. The effect of TNF blockade was observed mainly at late times suggesting that autocrine TNF prolongs the DC response to both β-glucan and LPS after the initial stimulus. This hypothesis is consistent with the reported ability of TNF to orchestrate in other cell types the temporal order of inflammatory gene expression [50], [51].

IL-1 antagonism down-regulated the expression of most late genes in β-glucan-exposed DCs. IL-1RA primarily reduced the expression of genes encoding inflammatory and immunoregulatory cytokines, antifungal and antibacterial molecules as well as cell adhesion and migration mediators. IL-1RA also promoted the expression of several antiviral response genes, similar to TNF neutralization. IL-10, IL-12, and the Th17-polarizing cytokines IL-6 and IL-23 were among the immunoregulatory factors critical for Th differentiation that were decreased by IL-1RA. IL-1 regulation of IL-23 production was previously reported [52], [53], but the molecular mechanisms remained unknown. Here, we show that IL-1 enhances the β-glucan-activated expression of *IL23A* as well as *IL6*, *IL10*, and *IL12B* in human DCs at the transcriptional level *via* IκB-ζ induction.

Genes encoding NF-κB family proteins are induced early by β-glucan. Dectin-1-mediated activation of NF-κB is critical for the production of IL-6, IL-10, IL-12, and IL-23 in human DCs [22]. However, IL-1 antagonism had no effect on β-glucan-induced gene expression and nuclear accumulation of NF-κB family components. The only exception was the *NFKBIZ* gene encoding IκB-ζ that was the earliest IL-1-regulated TF induced by β-glucan. Other early expressed TFs were not regulated by IL-1, whereas about half of the late induced TFs required the presence of autocrine IL-1 for optimal expression. The *NFKBIZ* gene is induced by MyD88- but not TRIF-associated receptors and IκB-ζ is indispensable for the induction of *IL6* and *IL12B* in LPS-treated murine DCs [35]. We reported that IκB-ζ is a key regulator of IL-6 production in LPS-stimulated human monocytes [34]. Here, we demonstrate by knockdown experiments in β-glucan-exposed DCs that IκB-ζ contributes to the expression of pro-inflammatory/immunoregulatory mediators (including *IL23A*, *IL6*, *IL10*, *IL12B*, but not *IL12A*), while it down-regulates several IFN-regulated genes, largely mirroring the effect of autocrine IL-1. Thus, we propose that IκB-ζ is the key factor by which the MyD88-dependent signaling by autocrine IL-1 supports the programming of human DCs mediating β-glucan responses and transcriptionally regulates inflammatory and immunoregulatory genes.

IκB-ζ has been shown to regulate the activity of nuclear NF-κB and CEBPβ to modulate, in a gene-specific manner, the expression of IL-6 and Lipocalin-2 [34], [54]–[56]. We now show that IL-1 regulates the DC response to β-glucan by promoting IκB-ζ, which in turn may cooperate with master TFs to transcriptionally activate target genes such as human *IL23A*. The ability of the human *IL23A* gene to be regulated by IκB-ζ contrasts with mouse data that have shown unimpaired *Il23a* expression in LPS-stimulated macrophages from *Nfkbiz* ^-/-^ mice [57]. We demonstrate that, similarly to *IL6* [34], IL-1-dependent gene transcription of *IL23A* is potentiated by IκB-ζ, which increases by several fold the activity of enhancing elements in the *IL23A* promoter upstream of the proximal NF-κB binding site required for activation of the promoter by IL-1β.

Innate receptor signaling programs APCs for the polarization of adaptive immune responses. IL-1 produced by APCs acts on T cells as a strong inducer of Th17 differentiation [58], [59]. We have shown that IL-1 released by DCs is primarily responsible for Th17 polarization induced by the yeast cell wall glucan, zymosan [26], [27]. IL-1 promotes IL-17 transcription in CD4^+^ T cells by inducing IκB-ζ that functionally interacts with RORγT [37]. We now show that an autocrine/paracrine IL-1-mediated positive feedback *via* IκB-ζ is also needed to program β-glucan-activated DCs for polarization of IL-17-producing Th cells. Thus, our findings highlight the dual role of DC-derived IL-1 in the Th17 response. It acts on both T cells and APCs through IκB-ζ which in turn regulates gene transcription by cooperating with master TFs.

De novo synthesis of IκB-ζ is efficiently, although transiently, induced by TLR ligands through MyD88, but it is poorly induced by ITAM signaling downstream of lectin receptors such as Dectin-1. Our data now demonstrate that the Th17-like type response promoted by β-glucan-activated DCs is dependent on IL-1 and on the IL-1/IκB-ζ-induced cytokines, IL-6 and IL-23. This suggests that the optimal programming of APCs by ligands for lectins or other ITAM-signaling receptors may require a feedback mechanism mediated by IL-1 inducing IκB-ζ *via* MyD88-dependent signaling. We show that β-glucan-induced IL-6 and IL-23 are dependent on IκB-ζ for optimal expression and that IκB-ζ is induced in both β-glucan- and LPS-stimulated DCs. However, only IL-6 was found to be released at similar levels by DCs stimulated by either β-glucan or LPS (Figure 1A). The analysis of the kinetics of gene expression of *IL6*, *IL23A*, and *NFKBIZ* suggested a possible explanation (Figure S6 in File S1). *IL6* is expressed at similar levels in presence of both stimuli (although earlier and more transient with LPS) with gene expression kinetics that rapidly follow and almost mimic those of *NFKBIZ* allowing the *IL6* gene to take full advantage of the presence of IκB-ζ for optimal expression. Unlike IL-6, *IL23A* is induced significantly only by β-glucan, later and in a more sustained manner, most probably facilitated by the delayed expression of the TF *NFKBIZ* in presence of β-glucan compared to LPS. This explanation is supported by the fact that the kinetic of *NFKBIZ* mRNA accumulation in DCs stimulated by β-glucan in the presence of IL-1RA mimics the kinetic of expression of *NFKBIZ* in LPS-treated cells and is associated with a decreased induction of the cytokine genes in response to β-glucan. Thus, the temporal expression of *NFKBIZ*, *IL6,* and *IL23A* explains the differential regulation of the Th17-polarizing cytokine genes *IL6* and *IL23A* in β-glucan- and LPS-activated DCs (Figure S6 in File S1).

In addition to autocrine/paracrine regulatory pathways, the activation of APCs is regulated by cytokines produced in the inflamed microenvironment by innate or immune cells. In particular, IFN-γ produced by NK or T cells contributes to the TLR agonist-promoted polarization of Th1 cells by affecting the differentiation of monocytes into DCs and by enhancing the production of IL-12 and IL-23 in TLR-activated DCs [7], [10], [26]. However, IFN-γ paradoxically inhibits the β-glucan-activated DC production of IL-23, but not IL-12 [26]. We now explain this paradoxical effect of IFN-γ by showing that it interferes with the IL-1/IκB-ζ pathway in human DCs exposed to β-glucan. IFN-γ has been shown to inhibit the production of IL-1β in both mouse and human cells by acting at the transcriptional level or by blocking IL-1β processing by the inflammasome, in an NO-dependent mechanism [49], [60]–[62]. We now show that, in β-glucan stimulated human monocyte-derived DCs, IFN-γ represses the production of IL-1β at the level of transcript accumulation and protein secretion as well as the ability of IL-1β to induce nuclear accumulation of IκB-ζ, without affecting the nuclear localization of other NF-κB components. Consistently, IFN-γ mimicked the effects of IL-1RA treatment and *NFKBIZ* knockdown on the β-glucan-associated gene signature in DCs. We propose that IFN-γ alters the balance between IL-23 and IL-12p70 in β-glucan-exposed DCs by acting mainly on their specific subunits. In fact, the expression of *IL23A* was dependent on IκB-ζ and blocked by both IL-1RA and IFN-γ, whereas the expression of *IL12A* was IκB-ζ-independent and induced by IFN-γ. Thus, IFN-γ reprograms DCs exposed to the CLR ligand β-glucan by altering, *via* the down-regulation of the IL-1/IκB-ζ axis, their pattern of cytokine production, in order to favor the induction of T cell-mediated immune responses with increased release of IFN-γ and IL-22, and diminished production of IL-17.

In conclusion, the present study reveals that β-glucan represents a potent activator of Th17-like-polarizing DCs not only because it activates ITAM-containing receptors such as Dectin-1, but also because of its ability to promote the release of IL-1β. IL-1β acts both at the APC level providing a potent positive feedback mechanism for the production of Th17-promoting cytokines such as IL-6 and IL-23 and, together with the latters, acts also on naïve T cells inducing IL-17 production and Th17 differentiation. Interesting, on both cell types IL-1β induces IκB-ζ that functions as a transcriptional cofactor by enhancing the production of relevant immunoregulatory molecules. The Th1 cytokine IFN-γ subverts this Th17-like polarization by blocking the IL-1β/IκB-ζ axis and therefore IL-6 and IL-23 production in β-glucan-activated DCs. Thus, these findings identify IL-1 and IFN-γ as unrecognized regulators of DC programming by β-glucan, revealing novel molecular networks that provide new insights into the differentiation of Th17 cells as well as new targets for the modulation of immune responses to Dectin-1 ligand-containing microorganisms such as fungi and mycobacteria.

## Materials and Methods

### Reagents and antibodies

The complete list of reagents and antibodies used in these studies is included in Materials and Methods S1 in File S2.

### Cells and cell cultures

Human monocyte-derived DCs were differentiated by 6/7-d cultures of elutriated monocytes from healthy donors with recombinant IL-4 (10 ng/ml; PeproTech) and GM-CSF (50 ng/ml; Leukine, Bayer). Human elutriated monocytes from healthy volunteers were from the Department of Transfusion Medicine, NIH Clinical Center. IFN-γ-primed monocyte-derived DCs were cultured overnight with IFN-γ. Human CD45RO^-^CD4^+^ naïve T lymphocytes were purified as previously described [26].

### Cytokine measurement by ELISA

Cytokines were measured using commercial ELISA kits for human TNF, IL-1α, IL-1β, IL-6, IL-10, IL-12p70, IL-17, IL-22, IL-23 (eBioscience), IL-12p40 (R&D Systems), and IL-18 (MBL International), and by an in-house ELISA for IFN-γ [63].

### RNA and cDNA preparation

Total RNA was extracted using the RNeasy Mini Kit (QIAGEN). Residual genomic DNA was digested twice. cDNA was prepared from 1 µg/sample of total RNA using random primers, dNTPs, and SuperScript II reverse transcriptase (Invitrogen).

### Real-Time qRT-PCR

Real-Time qRT-PCR was performed [64] using specific primers (Table S2 in File S1) for the detection of primary and mature transcripts. Data were analyzed with the StepOne software (Applied Biosystems) and standard curves, cycle threshold (Ct) values, and RNA input amount were used to calculate the copy number per ng of total RNA. GAPDH was used as endogenous control.

### Microarray

Total RNA from DCs was shipped on dry ice to GenUs BioSystems (Chicago, IL) where the microarray was performed.

### mRNA counting with nCounter

DCs were treated as indicated and lysed in RLT buffer (QIAGEN) supplemented with 1% β-mercaptoethanol. Cell lysates were hybridized with CodeSet and ProbeSet (NanoString) for 64 selected genes, for mRNA quantitation using the NanoString's nCounter Digital Analyzer.

### Chromatin immunoprecipitation (ChIP)

DCs were cultured as indicated and fixed with 1% formaldehyde, washed, and incubated for 10 min on ice in cold cell lysis buffer and in nuclear lysis buffer, both supplemented with protease and phosphatase inhibitors. Sheared chromatin was collected after sonication, diluted, and pre-cleared. Soluble chromatin was immunoprecipitated overnight at 4°C with Dynabeads protein G pre-absorbed with specific antibodies. Immunoprecipitated chromatin was eluted, treated with proteinase K (Invitrogen), and cross-links were reversed overnight at 65°C. DNA samples were analyzed by Real-Time qRT-PCR with the primers listed in Table S2 in File S1. The Ct value of each ChIP DNA sample was normalized to the corresponding input value and the % input DNA was calculated according to the SABiosciences guidelines.

### Western blotting

Cells were lysed on ice in hypotonic buffer supplemented with the appropriate inhibitors. The remaining nuclei were incubated in nuclear extraction buffer (Pierce) with all inhibitors. Cytoplasmic and nuclear extracts were separated on NuPAGE Novex 4-12% Bis-Tris Gel (Invitrogen). Proteins were then transferred onto Immobilon-P Transfer Membranes (Millipore). The membranes were blocked, blotted with specific primary antibodies, and then incubated with the appropriate secondary antibodies. The immunoblotted membranes were exposed to ECL Prime (GE Healthcare). The images were acquired using a ChemiDoc (Bio-Rad).

### siRNA transfection

DCs were transfected with a 10 nM pool of four specific *NFKBIZ* siRNAs or control siRNA (siGENOME; Dharmacon) using the INTERFERin siRNA transfection reagent (Polyplus Transfection). Gene expression analysis was performed with nCounter and the assessment of the knockdown efficiency by Western blotting.

### Plasmids and luciferase assay

Luciferase Reporter Assay System (Promega) was used according to the manufacturer's instructions for the measurement of the *IL23A* promoter activity in HeLa cells. Measurements were performed using a FLUOstar Omega (BMG Labtech).

### Statistics

For uncensored measurements, mean values were compared by paired *t*-tests or Wilcoxon signed-rank tests. A Tobit model analysis was used in presence of left-censored data. The effect of IL-1RA in Figure 5A was tested in a mixed-effects model. All the analyses were performed using the software package SAS 9.1.3, 2012 (SAS Institute Inc., Cary, NC, USA). n  =  number of donors, *p≤0.05, **p<0.005, ***p<0.0001, ns  =  not significant.

### Accession numbers

The microarray data are available in the Gene Expression Omnibus (GEO) database (http://www.ncbi.nlm.nih.gov/gds) under the accession number GSE42189.

### Ethics statement

Blood samples were collected from normal, healthy, adult donors at the National Institutes of Health Department of Transfusion Medicine under the protocol 99-CC-0168 approved by the National Institutes of Health Office of Human Subjects Research (Bethesda, MD, USA). A written informed consent was obtained and samples were provided anonymously to the laboratory.

## Supporting Information

File S1 **Supporting files. Figure S1, TNF and type I IFN modulate gene expression in both β-glucan- and LPS-activated DCs, whereas autocrine IL-1 selectively regulates the β-glucan response.** Human monocyte-derived DCs were cultured with (solid lines) or without (dashed lines) particulate β-glucan or LPS in the presence or absence of IL-1RA (25 µg/ml) or the indicated neutralizing antibodies for the times shown. mRNA levels in cell lysates were quantitated by NanoString's nCounter technology. The results (normalized mRNA counts) show the kinetics of mRNA accumulation of 12 selected genes in monocyte-derived DCs from one representative donor extracted from the data shown in Figures 3 and 4A. The vertical dashed lines across the panels mark the 12 h of culture as a visual help to follow the kinetic patterns of mature transcript accumulation. **Figure S2, Perturbation of autocrine IL-1 signaling in β-glucan activated-DCs does not change the mRNA stability of late immunoregulatory cytokines.** Human monocyte-derived DCs were left untreated (no stimulus) or stimulated for 10 h with particulate β-glucan in the absence or presence of IL-1RA (25 µg/ml). Actinomycin D (ActD) was added after 10 h of stimulation for the times shown. mRNA levels in cell lysates were quantitated by NanoString's nCounter technology. The results (normalized mRNA counts) are expressed as percentage of remaining mRNA counts from DCs activated for 10 h prior to ActD exposure and show the kinetics of mRNA accumulation of 4 selected genes in monocyte-derived DCs from one donor representative of three tested. **Figure S3, Perturbation of the inflammasome/IL-1 pathway decreases the release of late-induced cytokines by β-glucan-activated DCs.** (A) Human monocyte-derived DCs were cultured for 24 h in the presence or absence of particulate β-glucan and IL-1RA, control IgG, anti-IL-1α or anti-IL-1β as indicated. Protein secretion in the culture supernatants was measured by ELISA. Cytokine levels are expressed as percentage of cytokine released from DCs stimulated with β-glucan only. Results are mean ± SEM, n = 3 to 6. Statistics: paired t-test. (B) Human monocyte-derived DCs were cultured for 24 h with or without particulate β-glucan and Caspase 1 or Caspase 8 inhibitors or the solvent DMSO. Protein secretion in the culture supernatants was measured by ELISA. Cytokine levels are expressed as percentage of cytokine released from DCs stimulated with β-glucan + DMSO. Results are mean ± SEM, n = 6 to 8. Statistics: paired t-test. N.D.: not done. **Figure S4, IκB-ζ increases the IL-1β-dependent activity of a human** ***IL23A*** **promoter construct deleting the AP-1 site but containing the proximal NF-κB site.** HeLa cells were transfected with a basic pcDNA3.1 plasmid, pcDNA(Basic), or a pcDNA(*NFKBIZ*) along with the indicated pGL3 luciferase reporter vectors under the control of different lengths of the human *IL23A* promoter. At 24 h after transfection, cells were treated with IL-1β. Luciferase activity was read in cell lysates after 48 h of stimulation. Data were normalized to TK-*Renilla* activity and expressed relative to the normalized activity of the Basic vector. Results are mean ± SEM, n = 3. **Figure S5, IFN-γ does not alter the surface expression of IL-1R1 and IL-1RAcP in human DCs.** Surface expression of IL-1R1 and IL-1RAcP in IFN-γ-primed (red) or unprimed (blue) human monocyte-derived DCs was analyzed by flow cytometry on freshly prepared cells or after 4 h culture in the presence or not of β-glucan (filled histograms: isotype control; open histograms: specific antibody; values are MFI). Results are from one donor representative of three tested. **Figure S6, The temporal expression of** ***NFKBIZ*** **,** ***IL6*** **, and** ***IL23A*** **explains the differential regulation of** ***IL6*** **and** ***IL23A*** **in β-glucan- and LPS-activated DCs.** Human monocyte-derived DCs were cultured in the presence of particulate β-glucan with or without IL-1RA (25 µg/ml) or LPS as indicated. Gene expression data are from the experiment of Figure 4A and results shown are average of normalized mRNA counts from 8 different donors. **Table S1, List of immunoregulatory genes relevant for DC functions and inflammation selected on the basis of the microarray data for NanoString analysis. Table S2, Primer sequences used in** **Figures 1** **,** **5** **,** **7** **, and Figure S4.**
(DOCX)

File S2 **Materials and Methods S1 and References S1.**
(DOCX)
