# Supplementary material for: Interleukin-1 and Interferon-γ Orchestrate β-Glucan-Activated Human Dendritic Cell Programming via IκB-ζ Modulation
Source: PLoS One. 2014 Dec 4;9(12):e114516. doi: 10.1371/journal.pone.0114516 (PMC4256441; doi:10.1371/journal.pone.0114516)
Supplement: File S1 — Supporting files. Figure S1, TNF and type I IFN modulate gene expression in both β-glucan- and LPS-activated DCs, whereas autocrine IL-1 selectively regulates the β-glucan response. Human monocyte-derived DCs were cultured with (solid lines) or without (dashed lines) particulate β-glucan or LPS in the presence or absence of IL-1RA (25 µg/ml) or the indicated neutralizing antibodies for the times shown. mRNA levels in cell lysates were quantitated by NanoString's nCounter technology. The results (normalized mRNA counts) show the kinetics of mRNA accumulation of 12 selected genes in monocyte-derived DCs from one representative donor extracted from the data shown in Figures 3 and 4A. The vertical dashed lines across the panels mark the 12 h of culture as a visual help to follow the kinetic patterns of mature transcript accumulation. Figure S2, Perturbation of autocrine IL-1 signaling in β-glucan activated-DCs does not change the mRNA stability of late immunoregulatory cytokines. Human monocyte-derived DCs were left untreated (no stimulus) or stimulated for 10 h with particulate β-glucan in the absence or presence of IL-1RA (25 µg/ml). Actinomycin D (ActD) was added after 10 h of stimulation for the times shown. mRNA levels in cell lysates were quantitated by NanoString's nCounter technology. The results (normalized mRNA counts) are expressed as percentage of remaining mRNA counts from DCs activated for 10 h prior to ActD exposure and show the kinetics of mRNA accumulation of 4 selected genes in monocyte-derived DCs from one donor representative of three tested. Figure S3, Perturbation of the inflammasome/IL-1 pathway decreases the release of late-induced cytokines by β-glucan-activated DCs. (A) Human monocyte-derived DCs were cultured for 24 h in the presence or absence of particulate β-glucan and IL-1RA, control IgG, anti-IL-1α or anti-IL-1β as indicated. Protein secretion in the culture supernatants was measured by ELISA. Cytokine levels are expressed as percentage o [file pone.0114516.s001.docx]

**Figure S1**

**
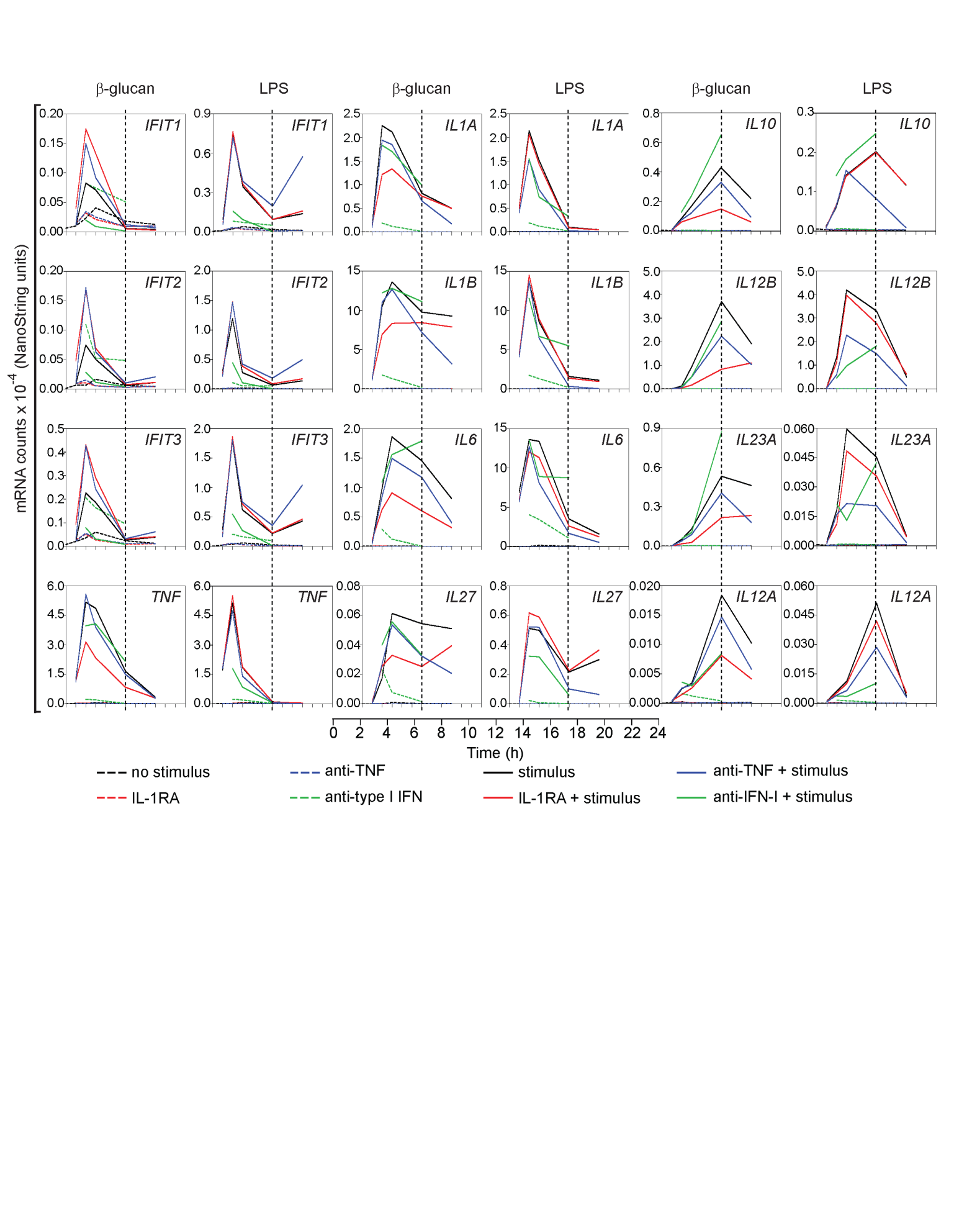
**

**Figure S2**

**
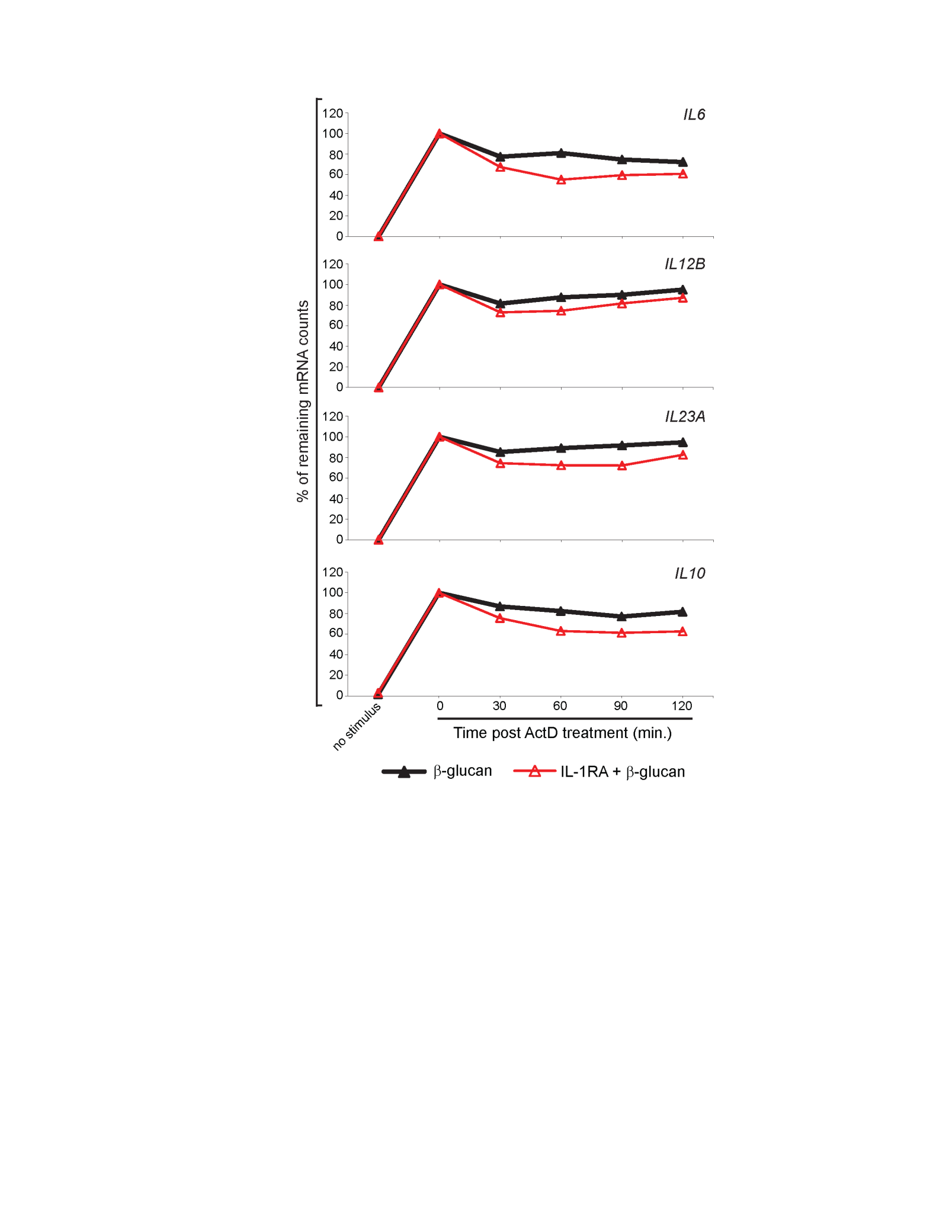
**

**Figure S3**

**
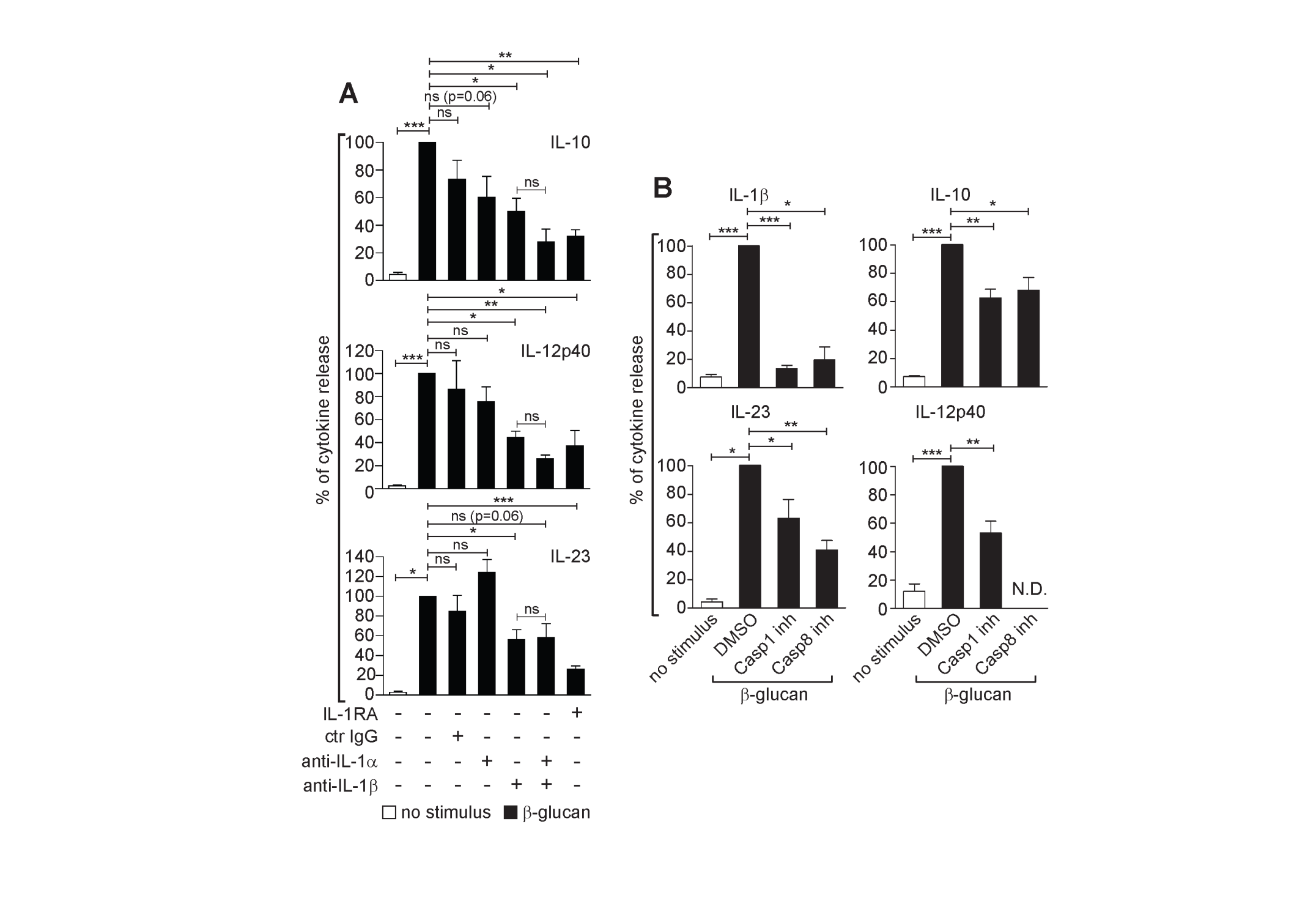
**

**Figure S4**

**
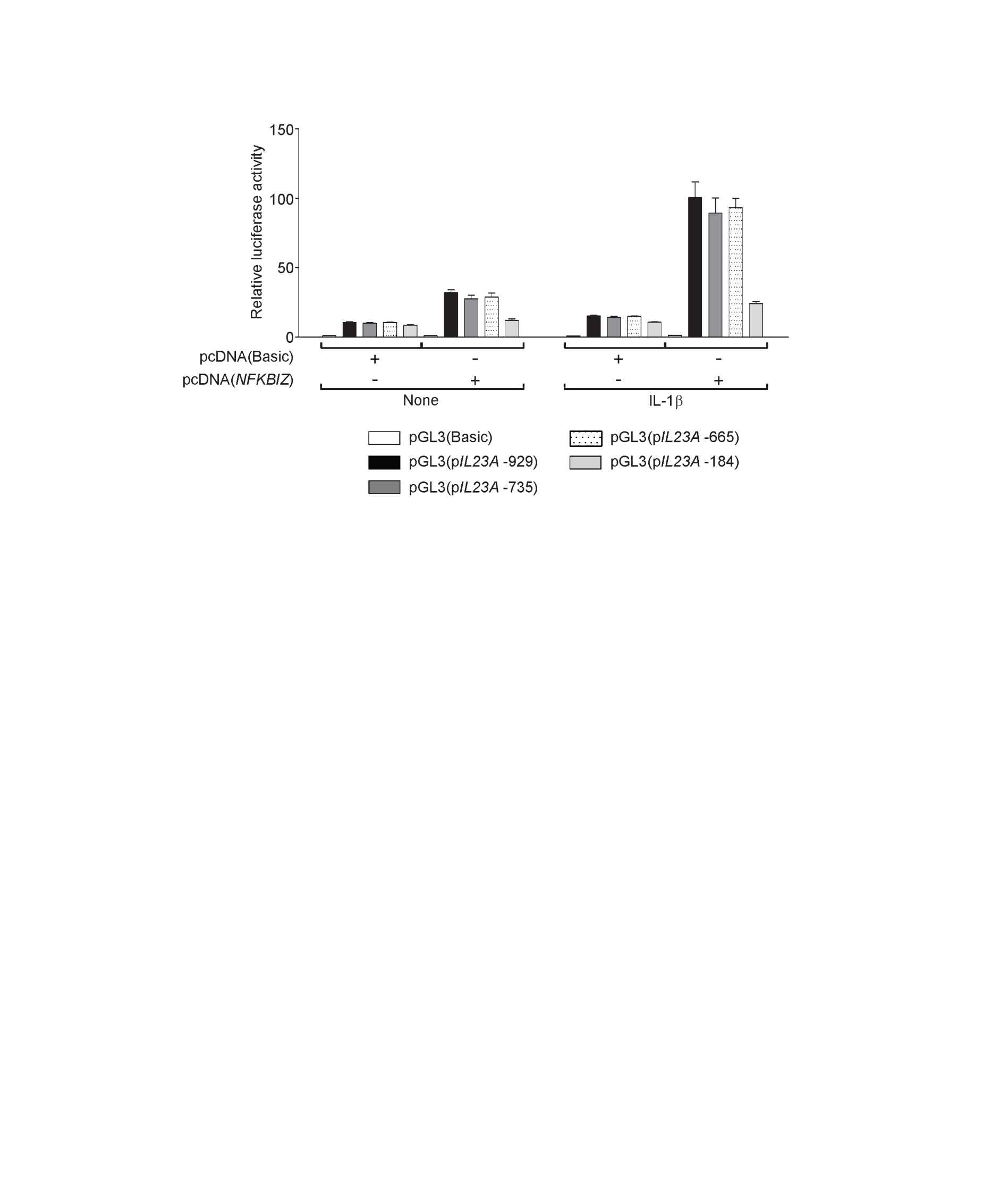
**

**Figure S5**

**
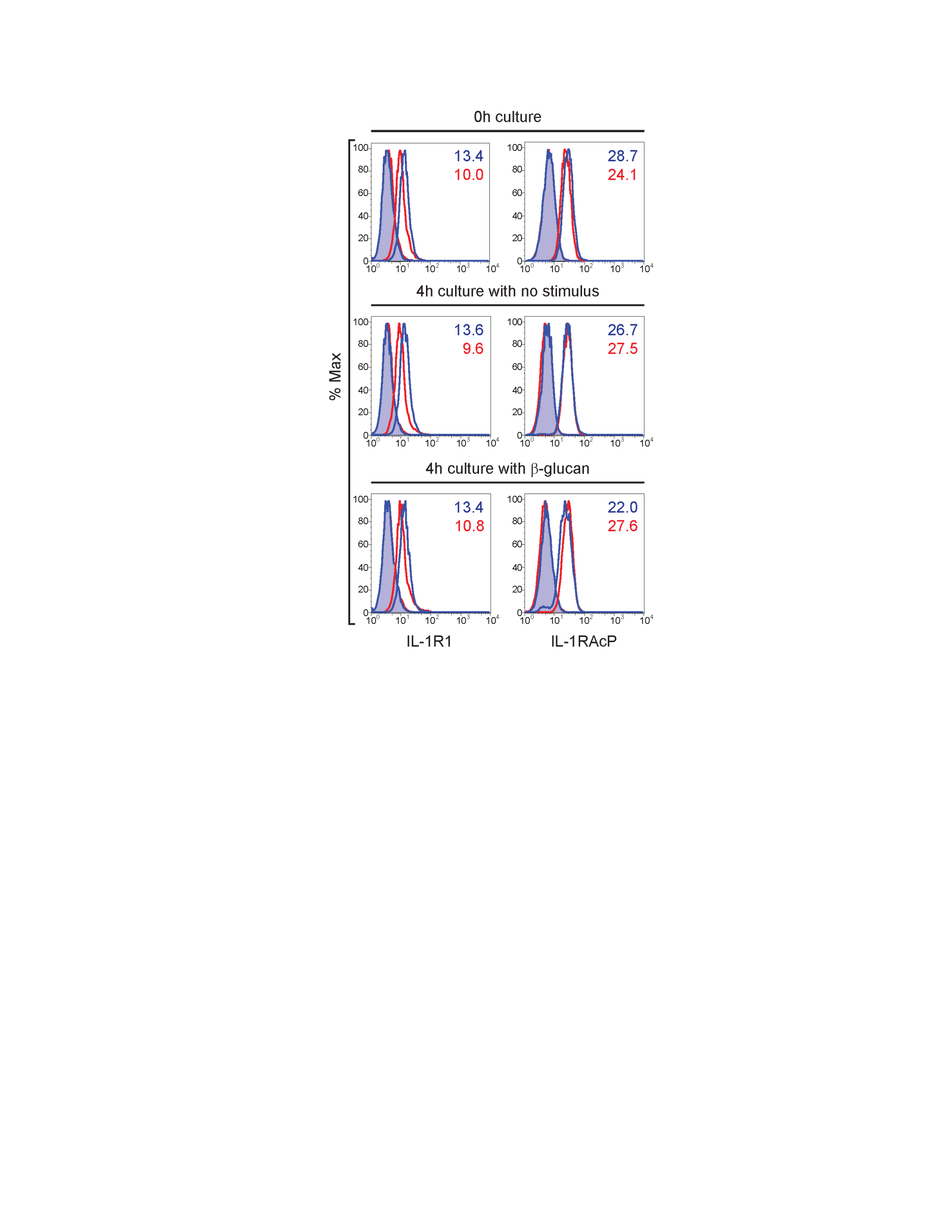
**

**Figure S6**

**
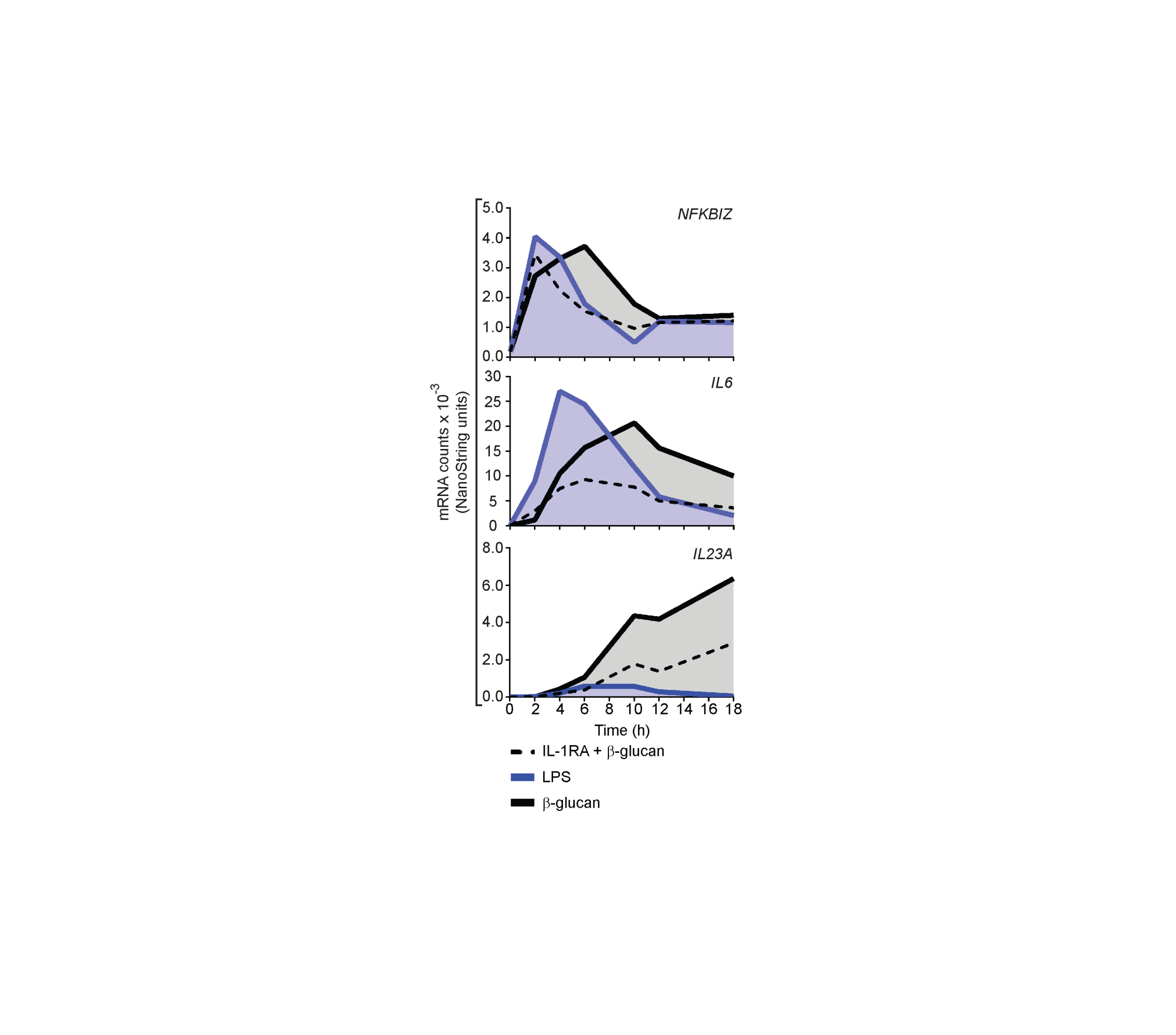
**

**Table S1. List of immunoregulatory genes relevant for DC functions and inflammation selected on the basis of the microarray data for NanoString analysis.**

|  | **Gene symbol** | **General function** | **Regulated by autocrine IL-1**  **(based on microarray)** |
| --- | --- | --- | --- |
| **4**  **Early**  **induced genes** | ***CCL2*** | **Factor** | **NO** |
|  | ***EGR1*** | **TR*** | **NO** |
|  | ***IFIT1*** | **Factor** | **NO** |
|  | ***IFIT2*** | **Factor** | **NO** |
|  | ***IFNB1*** | **Factor** | **YES** |
|  | ***MX1*** | **Other** | **NO** |
|  | ***MX2*** | **Other** | **NO** |
|  | ***NFATC1*** | **TR*** | **NO** |
|  | ***NFKBID*** | **TR*** | **NO** |
| **5**  **Early-late induced genes** | ***CASP1*** | **Other** | **NO** |
|  | ***CCL4*** | **Factor** | **NO** |
|  | ***CD83*** | **Other** | **NO** |
|  | ***CD86*** | **Other** | **NO** |
|  | ***CEBPB*** | **TR*** | **NO** |
|  | ***CLCF1*** | **Factor** | **NO** |
|  | ***CXCL1*** | **Factor** | **NO** |
|  | ***DRAM*** | **Other** | **NO** |
|  | ***EREG*** | **Factor** | **NO** |
|  | ***IFIT3*** | **Factor** | **NO** |
|  | ***IL1A*** | **Factor** | **NO** |
|  | ***IL1B*** | **Factor** | **NO** |
|  | ***IL1RN*** | **Other** | **NO** |
|  | ***IL6*** | **Factor** | **YES** |
|  | ***IL8*** | **Factor** | **NO** |
|  | ***IRF1*** | **TR*** | **NO** |
|  | ***IRF7*** | **TR*** | **NO** |
|  | ***IRF8*** | **TR*** | **NO** |
|  | ***MAP3K8*** | **Sign. Molec.**** | **NO** |
|  | ***NFKB1*** | **TR*** | **NO** |
|  | ***NFKBIA*** | **TR*** | **NO** |
|  | ***NFKBIZ*** | **TR*** | **YES** |
|  | ***PTX3*** | **Factor** | **NO** |
|  | ***SOCS3*** | **Other** | **NO** |
|  | ***SOD2*** | **Other** | **NO** |
|  | ***STAT3*** | **TR*** | **NO** |
|  | ***TLR2*** | **Receptor** | **NO** |
|  | ***TNF*** | **Factor** | **NO** |
|  | ***TNFAIP3*** | **Other** | **NO** |
| **6**  **Late**  **induced genes** | ***CCR7*** | **Receptor** | **NO** |
|  | ***CD38*** | **Other** | **YES** |
|  | ***CD80*** | **Other** | **NO** |
|  | ***CSF2*** | **Factor** | **NO** |
|  | ***CSF3*** | **Factor** | **YES** |
|  | ***CYP7B1*** | **Other** | **YES** |
|  | ***EBI3*** | **Factor** | **YES** |
|  | ***HES1*** | **TR*** | **YES** |
|  | ***IL10*** | **Factor** | **YES** |
|  | ***IL12A*** | **Factor** | **YES** |
|  | ***IL12B*** | **Factor** | **YES** |
|  | ***IL15*** | **Factor** | **NO** |
|  | ***IL15RA*** | **Receptor** | **YES** |
|  | ***IL1F9*** | **Factor** | **YES** |
|  | ***IL20*** | **Factor** | **YES** |
|  | ***IL23A*** | **Factor** | **YES** |
|  | ***IL27*** | **Factor** | **YES** |
|  | ***IDO1*** | **Other** | **NO** |
|  | ***ITGB8*** | **Other** | **YES** |
|  | ***MMP7*** | **Other** | **YES** |
|  | ***PTGS2*** | **Other** | **YES** |
|  | ***S100A8*** | **Other** | **YES** |
|  | ***S100A9*** | **Other** | **YES** |
|  | ***TGFA*** | **Factor** | **NO** |

***** Transcriptional regulator

****** Signaling molecule

**Table S2. Primer sequences used in Figures 1, 5, 7, and Figure S4.**

| **Gene symbol** | **Primer sequences**  **(primary transcripts)** | **Product size** | **Tm ºC** |
| --- | --- | --- | --- |
| ***IL6*** | F: tttcccaccatctttcctctt  R: GCTCTGGCTTGTTCCTCACT | 124 | 58 |
| ***IL10*** | F: tcctgcccttagGGTTACCT  R: TGAGGGTCTTCAGGTTCTCC | 145 | 58 |
| ***IL12B*** | F: CTAAGATGCGAGGCCAAGAA  R: GAAACCAGAGCAGTTTCACTCA | 121 | 58 |
| ***IL23A*** | F: GAGGGAGATGAAGAGACTACAAATG  R: cagccacatcccagtggt | 107 | 58 |
| **Gene symbol** | **Primer sequences**  **(mature transcripts)** | **Product size** | **Tm ºC** |
| ***TNF*** | F: AAGCCTGTAGCCCATGTTGT  R: TGAGGTACAGGCCCTCTGAT | 142 | 58 |
| ***IL1A*** | F: ATCAGTACCTCACGGCTGCT  R: CTTCATCTTGGGCAGTCACA | 150 | 59 |
| ***IL1B*** | F: CTGTCCTGCGTGTTGAAAGA  R: TGCTTGAGAGGTGCTGATGT | 177 | 58 |
| ***IL6*** | F: CCTTCCAAAGATGGCTGAAA  R: AGCTCTGGCTTGTTCCTCAC | 154 | 58 |
| ***IL10*** | F: CGCTGTCATCGATTTCTTCC  R: ATAGAGTCGCCACCCTGATG | 187 | 59 |
| ***IL12B*** | F: AGGCGAGGTTCTAAGCCATT  R: GCAGGTGAAACGTCCAGAAT | 148 | 59 |
| ***IL23A*** | F: ACTCAGTGCCAGCAGCTTTC  R: GGATCCTTTGCAAGCAGAAC | 190 | 59 |
| ***GAPDH*** | F: GAAGGTGAAGGTCGGAGTC  R: GAAGATGGTGATGGGATTTC | 226 | 56 |
| **Gene promoter** | **Primer sequences**  **(ChIP)** | **Product size** | **Tm ºC** |
| ***IL6*** | F: ACGACCTAAGCTGCACTTTTCC  R: GAGCCTCAGACATCTCCAGTCC | 213 | 60 |
| ***IL12B*** | F: TCTTGAAATTCCCCCAGAAGG  R: GGACGGAGAGTCCAATGGC | 164 | 60 |
| ***IL23A*** | F: CATCCCAGGCCTCTAGCC  R: GGTTTGGTTCCCTCAGTTGTTC | 239 | 60 |
| **Promoter** | **Primer sequences** | **Location*** |  |
| **p*IL23A* -929** | F: CGACTACCCAGAACTCCTGGGCTTCC  R: ACCGAGTTTGTAAGCTGCTTCCTGT | -929  +31 |  |
| **p*IL23A* -735** | F: GCCTATGAAATCTTGGGCCACTAGG  R: ACCGAGTTTGTAAGCTGCTTCCTGT | -735  +31 |  |
| **p*IL23A* -665** | F: GAGCCTTTCTAACCTCCACTGTGAGG  R: ACCGAGTTTGTAAGCTGCTTCCTGT | -665  +31 |  |
| **p*IL23A* -184** | F: TCCCAGGCCTCTAGCCACAGC  R: ACCGAGTTTGTAAGCTGCTTCCTGT | -184  +31 |  |

*****Relative to the start codon of the human *IL23A* gene
