# Supplementary material for: Interleukin-1 and Interferon-γ Orchestrate β-Glucan-Activated Human Dendritic Cell Programming via IκB-ζ Modulation
Source: PLoS One. 2014 Dec 4;9(12):e114516. doi: 10.1371/journal.pone.0114516 (PMC4256441; doi:10.1371/journal.pone.0114516)
Supplement: File S2 — Materials and Methods S1 and References S1. (DOCX) [file pone.0114516.s002.docx]

**Materials and Methods S1**

**Reagents and antibodies**

For DC stimulation we used the following reagents: β-glucan from baker’s yeast (10 μg/ml; Sigma-Aldrich), ultrapure LPS from *E. coli* strain 0111:B4 (100 ng/ml; InvivoGen), and recombinant human IL-1β (10 ng/ml; PeproTech). For DC priming we used recombinant human IFN-γ (500 IU/ml; Actimmune, InterMune). In some experiments Actinomycin D (4-10 μM; Sigma-Aldrich) was added to the cell cultures for blocking the transcript elongation.

Different sources of β-glucan (including β-glucan particles from Sigma, WGP 3-6 from Biothera, and Curdlan from Wako) were compared. β-glucan particles from Sigma contained >65% β-glucan (1,3/1,6) and no mannose. Polymyxin B did not affect DC activation by the Sigma β-glucan, excluding the Endotoxin contamination of this reagent. β-glucan from Sigma also triggered transient tyrosine phosphorylation in RAW macrophages transduced with Dectin-1, but not those transduced with Dectin-2, suggesting exclusive binding of Dectin-1 by this reagent. RAW cell lines were the gift of Dr. Philip Taylor, Cardiff University [[1](#_ENREF_1),[2](#_ENREF_2)]. The different sources of β-glucan, used at equivalent concentration, induced similar cytokine production in human monocyte-derived DCs, although the β-glucan from Sigma stimulated the greatest production of IL-23. Different doses of β-glucan from Sigma (from 0.1 to 20 μg/ml) were tested in preliminary experiments. 10 μg/ml was chosen as optimal concentration for its capacity to induce, in human monocyte-derived DCs, the highest cytokine production with the lowest cytotoxicity.

For neutralization experiments we treated the cells, 1 h before stimulation, with: recombinant human IL-1RA (at the concentrations indicated in the figure legends; Kineret, Amgen), anti-IL-1α (50 μg/ml; PeproTech), anti-IL-1β AS10 and control IgG 107.3 (50 μg/ml; BD), Caspase 1 (Ac-YVAD-CMK) and Caspase 8 (ZIE(OMe)TD(OMe)-FMK) inhibitors (50 μM; Calbiochem), anti-TNF B154.2 (1:200; [[3](#_ENREF_3)]), or a mixture of anti-IFN-α, anti-IFN-β, and anti-IFN-α/β receptor chain 2 (1.5:100; PBL Biomedical Laboratories). For chromatin immunoprecipitation experiments we used: anti-RNAPII CTD4H8 and control IgG (1 μg per immunoprecipitation; Millipore). For Western blotting we used the primary antibodies: anti-MAIL/IκB-ζ (1:3,000; [[4](#_ENREF_4)]), anti-p65/RelA 1207, anti-p50/p105 1141, and anti-p52/p100 1267 (1:1,000; [[5](#_ENREF_5)]), anti-c-Rel 265 (1:3,000; [[5](#_ENREF_5)]), anti-RelB 1319 (1:1,000; [[6](#_ENREF_6)]), anti-Lamin A/C 14 (0.5 μg/ml; Millipore), and anti-Actin C4 (1:20,000; Millipore), and the secondary antibodies: HRP-conjugated anti-rabbit IgG and anti-mouse IgG (1:25,000; Amersham). The antibodies used for flow cytometry were: anti-CD14-FITC M5E2, anti-CD1a-FITC HI149, and FITC-conjugated isotype controls (at the concentrations indicated in the manufacturer’s protocol; BD), anti-IL-1R1-PE, anti-IL-1RAcP/IL-1R3-PE 89412, and PE-conjugated isotype controls (at the concentrations indicated in the manufacturer’s protocol; R&D Systems).

**Cells and cell cultures**

Freshly isolated human monocytes were obtained from the Department of Transfusion Medicine, NIH Clinical Center, as elutriated product from apheresis collections of mononuclear cells from normal donors. Alternatively, elutriated monocytes from healthy volunteers were isolated from peripheral blood mononuclear cells (PBMCs) collected as previously described [[7](#_ENREF_7)]. Human monocyte-derived DCs were differentiated by 6/7-d cultures of elutriated monocytes from healthy donors with recombinant IL-4 (10 ng/ml; PeproTech) and GM-CSF (50 ng/ml; Leukine, Bayer), in antibiotic-free RPMI 1640 medium containing 10% heat-inactivated, low-endotoxin FCS and 2 mM L-glutamine. Monocyte-derived DCs were assessed by flow cytometry for the surface expression of CD1a and the lack of CD14. IFN-γ-primed DCs were obtained by priming human monocyte-derived DCs with IFN-γ the night before the end of the 6/7-d period of differentiation. At the beginning of each experiment, unprimed and IFN-γ-primed DCs were extensively washed to remove the excess of growth cytokines and IFN-γ, resuspended in fresh medium, and then treated as indicated in the figure legends. Human CD45RO^-^CD4^+^ naïve T lymphocytes were purified as previously described [[7](#_ENREF_7)] and cultured at 42,000 cells/well with plastic-bound anti-CD3 (5 μg/ml; Immunological Science) and soluble anti-CD28 (1:10,000) in U bottom, 96-well plates (Corning Life Sciences) in the presence of the DC supernatants indicated in the figure legends. Alternatively, as control, naive CD4^+^ T lymphocytes were polarized to Th17, Th22, and Th1 cells as follow: Th17 with recombinant human IL-1β (12.5 ng/ml; PeproTech), IL-6 (30 ng/ml; PeproTech), and IL-23 (25 ng/ml; R&D Systems); Th22 with only IL-6; Th1 with recombinant human IL-12 (5 ng/ml) and anti-IL-4 (5A4 mAb, 20 μg/ml + 4F2 mAb, 20 μg/ml). After 4 d of culture, polarized T lymphocytes were washed and stimulated with soluble anti-CD3 OKT3 (1:10,000) and PMA (10 ng/ml; Sigma-Aldrich) for further 18 h. HeLa cells were a kind gift of Dr. Xiaoxia Li (Cleveland Clinic Foundation, USA) and were maintained and transfected at 37°C and 5% CO_2_ in DMEM medium containing 10% heat-inactivated, low-endotoxin FCS, 2 mM L-glutamine, and 1% penicillin and streptomycin.

**RNA and cDNA preparation**

Total RNA was extracted using the RNeasy Mini Kit (QIAGEN). Residual genomic DNA in total RNA was digested twice, first during the RNA isolation using the RNAse-free DNAse Set (QIAGEN), and then with the DNAse I Amp Grade (Invitrogen) on extracted RNA. RNA concentration and purity were determined by measuring the OD at 260 nm, the 260/280 and the 260/230 ratios by NanoDrop (Celbio). cDNA was prepared from 1 μg/sample of total RNA using random primers, dNTPs, and SuperScript II reverse transcriptase (Invitrogen). In preliminary experiments, control reactions without reverse transcriptase were performed to verify by PCR the absence of genomic DNA contamination in the starting RNA samples.

**Real-Time qRT-PCR**

Real-Time qRT-PCR was performed [[8](#_ENREF_8)] using specific primers (Table S2 in File S1) for the detection of primary and mature transcripts. All primers were designed with the software Primer3 or Primer-BLAST starting from gene-specific sequences submitted to Ensembl and UCSC Genome Browser. The forward and reverse primers for each primary transcript were designed one in an exon and the other one in the adjacent intron. The primers for mature transcripts were designed in two different exons. The unstable nature of the unspliced primary transcripts was tested by determining their half-life after inhibition of transcription elongation with Actinomycin D and confirmed to be much shorter than 30 minutes compared with several hours for the corresponding spliced mature transcripts. The gene specificity of the primer sequences was verified in BLAST. Standard curves for the absolute quantification were created according to the Applied Biosystems guidelines. Data were analyzed with the StepOne software (Applied Biosystems) and standard curves, cycle threshold (Ct) values, and RNA input amount were used to calculate the copy number per ng of total RNA. GAPDH was used as endogenous control.

**Microarray**

Total RNA from DCs was shipped on dry ice to GenUs BioSystems (Chicago, IL) where the microarray was performed. Quality and quantity of the total RNA were assessed using an Agilent Bioanalyzer (Agilent Technologies). Single and double stranded cDNA were prepared starting from the total RNA samples. Double stranded cDNA was converted into cRNA target, whose quality was tested with the Bioanalyzer. The cRNA was subsequently fragmented to uniform size and hybridized to Agilent-014850 Whole Human Genome Microarray 4x44K G4112F (Probe Name version) arrays according to the manufacturer’s protocols. Slides were washed and scanned on an Agilent G2565 Microarray Scanner. Data were extracted with the Agilent Feature Extraction software and analyzed with Partek Genomics Suite software. Quantile-normalized, log2 – transformed data were used for downstream statistical analysis. We used two-way ANOVA to identify genes specific for the β-glucan response, using time points and treatment groups as variables. Genes with q<0.05 and >|2| fold change in any of the groups relative to untreated cells were used in further analysis. The list of β-glucan significant genes was used to filter the dataset and IL-1-dependent genes were identified in a similar way. Gene groups were identified as following: “Early” genes (*i*): *i*(t_4h_) >|2| *i*(t_0h_) & *i*(t_4h_) >|2| *i*(t_12h_); “Early-Late” genes: *i*(t_4h_) >|2| *i*(t_0h_) & *i*(t_4h_) <|2| *i*(t_12h_); “Late” genes: *i*(t_12h_) >|2| *i*(t_0h_) & *i*(t_12h_) >|2| *i*(t_4h_). Gene Ontology, TRANSFAC, and Ingenuity databases were respectively used for the analysis of the pathways associated with the genes induced by β-glucan, for the prediction of their transcription factors, and to identify potential regulators of the response of human DCs to β-glucan. Prediction of the TF binding sites was done using sequences -1000, +100 TSS. After identification of GO, canonical pathway and tentative TFs, we constructed networks in Cytoscape 2.8.2 (www.cytoscape.org) using genes and related GO terms and predicted TFs as nodes. Size of the nodes (only GO, Canonical pathways and TFs) was coded according to the inverse log10 p-value. Superclusters of related GO/canonical pathways terms or TFs with genes were identified using ClusterOne plugin of the Cytoscape using default settings. Clusters were then named according to the dominant group of the pathways after careful manual examination of the related genes and pathways/TF groups.

**mRNA counting with nCounter**

DCs were treated as indicated and lysed at the concentration of 2000 cells/μl in RLT buffer (QIAGEN) supplemented with 1% β-mercaptoethanol. 5 μl of cell lysate per sample were used for the analysis. Cell lysates were hybridized with CodeSet and ProbeSet (NanoString) for 64 selected genes, for mRNA quantitation using the NanoString’s nCounter Digital Analyzer. *PPIB* was chosen as a housekeeping gene for the normalization of the data. Data analysis was performed with Partek Genomics Suite software. Briefly, normalized log2-transformed data were used to perform t-tests between the groups mentioned in the result section. Significant genes (FDR<0.1) were used to filter the data and construct heat maps (blue-white-red color coded). For the visualization of the fold inhibition/stimulation by different treatments (IL1RA, anti-TNF, and anti-type I IFN) we used fold-inhibition/stimulation results calculated as the ratio between data from cells treated with β-glucan or LPS + IL-1RA or cytokine antibodies and data from cells treated only with β-glucan or LPS at every time point reported in the result section. Fold inhibition/stimulation were then visualized without any further mathematical manipulations in blue-yellow-red color coded heat maps. Full details on the NanoString’s nCounter technology are reported in [[9](#_ENREF_9)].

**Chromatin immunoprecipitation (ChIP)**

DCs were cultured as indicated and detached from the culture dishes using a Cell Dissociation Buffer enzyme-free (GIBCO). Fixed cells (1% formaldehyde) were incubated for 10 min on ice in cold cell lysis buffer (10 mM Tris-HCl pH8.0, 10 mM NaCl, 0.2% NP-40) and in nuclear lysis buffer (25 mM Tris-HCl pH8.0, 10 mM EDTA, 1% SDS), both supplemented with protease (Complete Mini, Roche) and phosphatase (Thermo) inhibitors. Cell lysates were then sonicated with a Bioruptor (Diagenode). Sheared chromatin was collected, diluted 10-fold in cold ChIP dilution buffer (10 mM Tris-HCl pH8.0, 1 mM EDTA, 125 mM NaCl, 0.6% NP-40, 0.04% SDS), supplemented with protease and phosphatase inhibitors, and pre-cleared with Dynabeads protein G (Invitrogen) pre-absorbed with normal mouse IgG. After removing a 1% aliquot for the input control, soluble chromatin was immunoprecipitated overnight at 4°C with rotation with Dynabeads protein G pre-absorbed with specific antibodies listed in the “Reagents and antibodies” section. Beads were washed with RIPA buffer (10 mM Tris-HCl, 1 mM EDTA, 1% Triton X-100, 0.1% SDS, 0.1% Na-Deoxycholate), RIPA buffer supplemented with 0.3 M NaCl, LiCl buffer (10 mM Tris-HCl, 1 mM EDTA, 250 mM LiCl, 0.5% NP-40, 0.5% Na-Deoxycholate), and TE buffer (10 mM Tris-HCl, 1 mM EDTA). ChIP and input DNA were purified with the QIAquick PCR Purification Kit (QIAGEN). Immunoprecipitated chromatin was eluted, treated with proteinase K (Invitrogen), and cross-links were reversed overnight at 65°C. DNA samples were analyzed by Real-Time qRT-PCR with the primers listed in Table S2 in File S1. The Ct value of each ChIP DNA sample was normalized to the corresponding input value and the % input DNA was calculated according to the SABiosciences guidelines.

**Western blotting**

Cells were cultured, detached using a Cell Dissociation Buffer enzyme-free (GIBCO), and lysed on ice in hypotonic buffer (25 mM Tris-HCl pH7.5, 1 mM MgCl_2_, 5 mM KCl, 0.3% NP-40) supplemented with protease (Complete Mini, Roche) and phosphatase (Thermo) inhibitors, and α_2_-Macroglobulin (Roche). Cell lysates were centrifuged at 500 X *g* for 5 min at 4°C and the supernatants containing the cytoplasmic protein fraction were collected. The remaining nuclei were incubated on a shaker for 20 min at 4°C in nuclear extraction buffer (Pierce) with all inhibitors. Nuclear lysates were centrifuged at 14,000 X *g* for 15 min at 4°C and the supernatants containing the nuclear protein fraction were collected. Protein extracts were quantified by BCA protein assay (Pierce) and equal protein amounts (5-10 μg) per lane were separated on NuPAGE Novex 4-12% Bis-Tris Gel (Invitrogen) at 110 V. The separated proteins were transferred onto Immobilon-P Transfer Membranes (Millipore) overnight at 4°C and 25 V. The membranes were blocked in 5% milk for at least 1 h at RT, blotted with specific primary antibodies for 1 h at RT, and then incubated with the appropriate secondary antibodies for 1 h at RT. The immunoblotted membranes were washed, and exposed to ECL Prime (GE Healthcare). The antibodies used are listed in the “Reagents and antibodies” section. The images were acquired using a ChemiDoc (Bio-Rad).

**siRNA transfection**

Human monocyte-derived DCs (0.6 X 10^6^ cells/ml) were plated in 12-well plates, in antibiotic-free RPMI 1640 medium containing 3% heat-inactivated, low-endotoxin FCS and 2 mM L-glutamine. Cells were transfected with 10 nM *NFKBIZ* siRNA (siGENOME SMARTpool; Dharmacon) or control siRNA (siGENOME Non-Targeting siRNA; Dharmacon) using the INTERFERin siRNA transfection reagent (Polyplus Transfection) according to the manufacturer’s instruction. 40 h after transfection (including the last 12 h of stimulation) DCs were harvested and lysed for the gene expression analysis. Gene expression analysis was performed with nCounter and the assessment of the knockdown efficiency by Western blotting.

**Plasmids and luciferase assay**

Different lengths of wild type or mutated *IL23A* 5’ upstream promoter fragments were generated by PCR using gene-specific forward and reverse primers listed in Table S2 in File S1. NF-κB-mutant promoter fragments were made by site-directed mutagenesis. PCR products were cloned into TOPO-TA vector (Invitrogen), and inserts were excised with *SacI/XhoI* and cloned into pGL3 (Promega) to obtain luciferase reporter vectors. The quality of all constructs was verified by sequencing. pcDNA(*NFKBIZ*) (MAIL pCDNA) was generated as previously described [[4](#_ENREF_4)]. Approximately 12 h prior to transfection, HeLa cells were plated at 1 X 10^4^ cells/well in 96-well plates. Cells were cotransfected using 0.5 μl/well of HilyMax transfection reagent (Dojindo) following the manufacturer’s protocol, and then stimulated as indicated in the legend of Figure 7B. For the transfection we used: pRL-TK Renilla luciferase control reporter vector (4 ng/well; Promega), pcDNA3.1 plasmids (16 ng/well), and pGL3 luciferase reporter vectors (80 ng/well). For the stimulation we used recombinant human IL-1β (50 ng/ml; PeproTech). Luciferase activity was measured in cell lysates after 48 h of stimulation using the Dual-Luciferase Reporter Assay System (Promega) according to the manufacturer’s instructions. Measurements were performed using a FLUOstar Omega (BMG Labtech).

**Flow cytometry**

Monocyte-derived DCs were stained with antibodies for 30 min at 4°C in Hanks' balanced salt solution containing 1% BSA, 0.1% NaN_3_, and 10% serum. Cells were analyzed with a FACSCalibur (BD).

**Statistics**

For uncensored measurements, mean values were compared by paired *t*-tests or Wilcoxon signed-rank tests. A Tobit model analysis was used in presence of left-censored data. The effect of IL-1RA in Figure 5A was tested in a mixed-effects model with donors as random effects, and with spatial covariance matrices to model correlation between measurements over time. Contrasts were then made to compare mean cytokine levels at specific time points, and area under the curve comparisons allowed mean cytokine comparisons simultaneously across time points. All the analyses were performed using the software package SAS 9.1.3, 2012 (SAS Institute Inc., Cary, NC, USA). n = number of donors, *p ≤ 0.05, **p < 0.005, ***p < 0.0001, ns = not significant.

**References S1**

1. Brown GD, Herre J, Williams DL, Willment JA, Marshall ASJ, et al. (2003) Dectin-1 Mediates the Biological Effects of β-Glucans. The Journal of Experimental Medicine 197: 1119-1124.

2. Suram S, Gangelhoff TA, Taylor PR, Rosas M, Brown GD, et al. (2010) Pathways Regulating Cytosolic Phospholipase A2 Activation and Eicosanoid Production in Macrophages by Candida albicans. Journal of Biological Chemistry 285: 30676-30685.

3. Cuturi MC, Murphy M, Costa-Giomi MP, Weinmann R, Perussia B, et al. (1987) Independent regulation of tumor necrosis factor and lymphotoxin production by human peripheral blood lymphocytes. J Exp Med 165: 1581-1594.

4. Seshadri S, Kannan Y, Mitra S, Parker-Barnes J, Wewers MD (2009) MAIL Regulates Human Monocyte IL-6 Production. The Journal of Immunology 183: 5358-5368.

5. Rice NR, MacKichan ML, Israël A (1992) The precursor of NF-κB p50 has IκB-like functions. Cell 71: 243-253.

6. Lyakh LA, Koski GK, Telford W, Gress RE, Cohen PA, et al. (2000) Bacterial Lipopolysaccharide, TNF-α, and Calcium Ionophore Under Serum-Free Conditions Promote Rapid Dendritic Cell-Like Differentiation in CD14+ Monocytes Through Distinct Pathways That Activate NF-κB. The Journal of Immunology 165: 3647-3655.

7. Gerosa F, Baldani-Guerra B, Lyakh LA, Batoni G, Esin S, et al. (2008) Differential regulation of interleukin 12 and interleukin 23 production in human dendritic cells. J Exp Med 205: 1447-1461.

8. Salcedo R, Worschech A, Cardone M, Jones Y, Gyulai Z, et al. (2010) MyD88-mediated signaling prevents development of adenocarcinomas of the colon: role of interleukin 18. J Exp Med 207: 1625-1636.

9. Geiss GK, Bumgarner RE, Birditt B, Dahl T, Dowidar N, et al. (2008) Direct multiplexed measurement of gene expression with color-coded probe pairs. Nat Biotechnol 26: 317-325.
